# Weights for ordinal analyses of the modified Rankin Scale in stroke trials: A population-based cohort study

**DOI:** 10.1016/j.eclinm.2020.100415

**Published:** 2020-06-15

**Authors:** Aravind Ganesh, Ramon Luengo-Fernandez, Sarah T. Pendlebury, Peter M. Rothwell, Oxford Vascular Study

**Affiliations:** Centre for Prevention of Stroke and Dementia, Nuffield Department of Clinical Neurosciences, University of Oxford, Level 6, West Wing, John Radcliffe Hospital, Oxford OX3 9DU, United Kingdom

**Keywords:** All Cerebrovascular disease/Stroke, Prognosis, Cohort studies, Clinical trials Methodology/study design, Outcome research

## Abstract

**Background:**

Ordinal/shift analyses of ordered measures like the modified Rankin Scale(mRS) are underused as primary trial outcomes for neurological disorders – despite statistical advantages – potentially hindered by poor clinical interpretability versus dichotomies, and by valuing state-transitions equally (linear scale). Weighted ordinal analyses incorporating step-changes at key transitions might have greater statistical validity and clinical applicability.

**Methods:**

In a prospective population-based cohort of ischaemic stroke (Oxford Vascular Study, recruited 2002-2014), we stratified 5-year outcomes of death, dementia, and/or institutionalization, health/social-care costs, and EuroQol-derived quality-adjusted life-expectancy(QALE) by 3-month mRS. We compared root-mean-square errors(RMSEs) from linear regressions for these outcomes with the mRS coded as a linear scale versus incorporating a spline at transitions 1-2, 2-3, or 3-4. We derived 3-month mRS weights for probability of 5-year death/dementia/institutionalization using age/sex-adjusted logistic regressions, and cost and QALE weights from 1000-bootstraps. We applied these weights to analyse recent trials of thrombectomy for acute ischaemic stroke.

**Findings:**

Among 1,607 patients, a non-linear (S-shaped) relationship was observed between 3-month mRS and each 5-year outcome, with RMSEs 18-73% lower using a spline at mRS 2-3 versus a linear representation. Age/sex-adjusted probability weights for 5-year death/dementia/institutionalization were: mRS 0=0.19; 1=0.27; 2=0.41; 3=0.73; 4=0.77; 5=0.94 (mRS 6=1 by definition). Similar trends were seen with costs; estimated 5-year QALEs were: mRS 0=3.88; 1=3.49; 2=3.01; 3=1.87; 4=1.30; 5=0.06; 6=0. Results were similar stratifying by age/sex, and excluding pre-morbidly disabled patients. Using a weighted ordinal approach, estimates of thrombectomy impact were more favourable than estimates with dichotomous approaches, 5-year cost reductions being 29% higher than with 0-2/3-6, and over three-fold higher than with 0-1/2-6 dichotomy.

**Interpretation:**

Our findings favour weighting the mRS in ordinal analyses for stroke and other neurological disorders, as state-transitions differ in clinical prognosis, quality-of-life, and costs. These weights could also be used for prognostication and cost-effectiveness analyses.

**Funding:**

Wellcome Trust, Wolfson Foundation, NIHR Oxford Biomedical Research Centre, Rhodes Trust.

**Research in context**

**Evidence before this study**

We searched PubMed for articles on the analysis of ordinal outcome scales published between 1-Jan-1993 and 1-Nov-2019, combining the terms “ordinal”, “dichotomous”, or ”dichotomization” with “outcome” and “trial”, and for studies of the modified Rankin Scale (mRS) for stroke by adding the terms “stroke” and “Rankin Scale”. Prior studies such as those by OAST (Optimising Analysis of Stroke Trials Collaboration) have demonstrated many pitfalls of dichotomizing commonly-used ordinal outcome scales like the mRS and have shown the statistical superiority of approaches retaining the full range of scores. However, ordinal analyses continue to be poorly adopted, hindered by poor clinical interpretability and treatment of all state-transitions as equal despite the non-linearity of many scales. The utility-weighted mRS has been proposed as an alternative for stroke trials, but weighting scales using cross-sectional quality-of-life ratings can be too subjective, particularly when not derived from the patients themselves.

**Added value of this study**

In this large population-based prospective cohort study of ischaemic stroke, we demonstrated a non-linear, S-shaped relationship between the 3-month mRS and 5-year clinical and health economic outcomes, and then derived probability weights for death/dementia/institutionalization as well as cost and quality-adjusted life expectancy (QALE) weights for each 3-month mRS grade. Applying these weights to recent trials of thrombectomy for acute ischaemic stroke, we demonstrated that this prognosis-weighted ordinal analysis of the mRS does not compromise the statistical efficiency of unweighted ordinal approaches and better demonstrates the expected long-term persistence/magnification of treatment effects than dichotomous or unweighted ordinal analyses.

**Implications of all the available evidence**

Cohort-derived natural history data for objective clinical and health-economic endpoints can be used for prognosis-weighted analyses of ordinal outcomes like the mRS in clinical trials. By acknowledging that certain state-transitions are more valuable than others whilst retaining the full range of the scale, a weighted approach can facilitate ordinal analyses that identify clinically meaningful treatment differences. Prospective cohort studies of other medical conditions can derive similar probability weights for commonly used ordinal scales to adopt this clinically grounded paradigm for ordinal analysis.

Alt-text: Unlabelled box

## 1 Introduction

Ordinal scales are commonly used in clinical trials [1,2]. The modified Rankin Scale (mRS) is used in over 20 neurological conditions (Appendix 1 and 2) and is the favoured primary outcome measure in stroke trials, partly due to ease of administration, reproducibility, and minimal floor or ceiling effects [3,4]. However, analyses of trials using such measures are often hampered by dichotomous approaches, which result in information loss, risk ignoring bi-directional effects, and often require larger samples than ordinal approaches (examining all outcome levels). Dichotomies also promote exclusion of pre-morbidly disabled patients (e.g. mRS≥2 pre-stroke) who cannot contribute positively to dichotomy-defined treatment effects [5,6].

Despite these pitfalls of dichotomization, adoption of ordinal analyses remains poor [7,8]. Although there has been a trend in recent years towards the use of ordinal analyses, dichotomous approaches continue to be favoured as the primary outcome by many high-profile trials [9,10]. One reason may be the poor clinical interpretability of conventional ordinal approaches, providing outputs like p-values or common odds-ratios without intuitive effect sizes [11]. Such approaches also value state-transitions equally. However, previous data on mRS-stratified quality-of-life ratings and 90-day home-time post-stroke suggest steeper slopes between grades 3-5 versus 0-2, with the greatest step-change potentially seen from mRS=2 to mRS=3, the conventional dichotomy cut-off, although this non-linearity has not been systematically tested [12,13]. If the mRS is indeed non-linear in relation to key outcomes, this would favour weighting the scale in ordinal analyses to reflect real-world differences between health-states. This approach would capture changes across the scale's range like conventional ordinal analyses, and acknowledge the greater clinical importance of certain state-transitions, like dichotomous analyses.

If an ordinal scale is to be weighted, deriving relevant weights from high-quality data is crucial. One example is the utility-weighted mRS (UW-mRS),[14] in which weights for each grade were obtained by averaging values from stroke/TIA survivors,[12] and disability weights from a professional panel [15]. Utilities and disability-weights are, however, limited by ultimate derivation from unaffected individuals, and vary by beliefs and sociodemographic factors [16]. On the other hand, long-term outcomes like death, institutionalization, or disease-specific endpoints (like post-stroke dementia) may be less vulnerable to bias and reflect natural history. Weighting ordinal scales based on the probability of such outcomes in prospective cohorts could also allow estimation of whether treatment effects will be magnified or eroded over time. Quality-adjusted life expectancies (QALEs, quality-adjusted life-years/QALYs expected over a time-period for a health-state) could also temper uncertainties of utility-weights with the relative definitiveness of mortality data. Together with cost weights, QALE weights could also facilitate cost-effectiveness analyses. Therefore, we examined the non-linearity of the relationship of 3-month mRS to 5-year death, institutionalization, dementia, costs, and QALE in ischaemic stroke patients in the Oxford Vascular Study (OXVASC), and used this data to derive weights for the mRS.

## 2 Methods

The OXVASC population comprises 92,728 individuals registered with 100 general practitioners(GPs) in 9 Oxfordshire practices. Study methods have been published [17]. Recruitment is ongoing since April-2002. Near-complete ascertainment of suspected strokes is achieved using overlapping “hot” and “cold” pursuit methods [18]. Patients with ischaemic stroke recruited from April-2002 to March-2014 were included. Patients were assessed urgently by study clinicians. Stroke was diagnosed per the World Health Organization definition [19]. All cases were reviewed with senior neurologist PMR. Patients had in-person follow-up with a study nurse/physician in clinic or at home at 1-month, 3-months, 6-months, 1-year, and 5-years. Patients who moved away received telephone follow-up. Additional information was obtained from carers in patients with impaired cognition or speech. Raters received mRS training via an instructional DVD with written materials by the University of Glasgow, used in prior trials [20]. At follow-up, patients/carers were asked about living arrangements. Medical records were also reviewed to identify dates of admission to nursing or residential care homes (institutionalization).

Deaths were recorded via death certificates, coroners’ reports, and the National Health Service (NHS) Central Register. Health and social-care resource use was obtained from the date of the first stroke in study-period (“index” stroke) until 5-years post-stroke or 15-May-2017, whichever was first. Methods for collecting resource use have been reported [21]. Briefly, patients’ records from the Oxford University Hospitals Trust were reviewed for any emergency visit/transport, outpatient-care visit, day case, or hospitalisation. For hospitalisations, dates of admission, discharge, and inter-ward transfers were recorded. Resource use was valued using the NHS schedule's unit costs [22]. Institutionalized days were estimated as the difference between date of 5-year follow-up or death, whichever was earliest, and date of institutionalization, and costed using the cost/week in private nursing homes, £795($1,145) in 2016 [23]. Costs were converted from UK pounds sterling(£) to US dollars($) using the 2016 rate of purchasing power parities ($=£0.694, http://stats.oecd.org/). It should be noted that the five-year mortality and cost data for this cohort, stratified by 3-month mRS, were previously published in an analysis comparing unweighted ordinal and dichotomous approaches for analysing the mRS; however, this did not include the mRS-stratified data for follow-up years 2 through 4, and we did not previously test the non-linearity of these data or derive probability weights as outlined below [6].

The methodology of dementia diagnosis in OXVASC, with overlapping methods of cognitive testing, interview-based assessments of patients and carers, and searches of medical records, has been reported (see **Appendix 3**) [24]. Quality-of-life assessment methods have been described [25]. At 6-month, 1-year, and 5-year follow-ups, quality-of-life was assessed using the EuroQol EQ-5D-3 questionnaire, a valid post-stroke measure enquiring about problems (none, some, unable/extreme) in five attributes: mobility, self-care, usual activities, pain/discomfort, and anxiety/depression [12]. EQ-5D responses were converted into utilities using UK population tariffs derived using time-trade-off from 3,337 individuals,[14] with regression equations fitted to obtain tariffs for all 243 possible EQ-5D health states, ranging from -0.59 to 1 [26]. The mRS-stratified five-year institutionalization, dementia outcomes, and quality-of-life data for this cohort have not been previously published. The study was approved by the Oxfordshire Research Ethics Committee.

### 2.1 Statistical analyses

Analyses were censored at 15-May-2017 and performed using STATA 13.1. Outcomes of death, death/dementia, death/institutionalization, and death/dementia/institutionalization were plotted against 3-month mRS, with the composite outcomes intended to capture dual burdens of disability and mortality with mRS increments [27]. Mean censor-adjusted costs for 1-year through 5-years were estimated [28] by partitioning the study-period into smaller time-periods (by day), calculating total costs incurred for patients alive at the beginning of each time-period, weighting estimated costs of patients with complete data by the Kaplan-Meier sample average estimator, and summing over time-periods. Quality-adjusted survival curves were generated by plotting, against time, the product of mean utility at each follow-up and probability of surviving to that follow-up. This area-under-the-curve represents mean QALE between 3-months and 5-years. We did not perform multiple imputation of missing EQ-5D data, since a recent analysis of post-stroke/TIA utilities in OXVASC found little change in results upon such imputation [25].

To test whether the relationship between 3-month mRS and 5-year outcomes was better described as non-linear versus linear (meriting weighting), we obtained theoretical mRS weights using a linear spline with knots at mRS=2 and mRS=3 and coefficients/slopes of 1, 4, and 2 for the resulting segments. This incorporated steeper slopes in the latter portion of the scale and a step-change between 2 and 3 as suggested in prior mRS-stratified data (**Appendix 4**) [12,13]. To verify whether appropriate step-change placement was at 2-3, we also evaluated splines with knots at mRS=1 and mRS=2, or mRS=3 and mRS=4. We compared root-mean-square errors (RMSEs) from linear regressions of 5-year outcomes by 3-month mRS, coded as a numerical variable either with standard values (0-5) or these theoretical weights. Non-linear relationships would be better linearized by weighting the mRS and effectively stretching parts of the x-axis, thereby lowering RMSE versus standard plots.

To then obtain data-derived mRS weights, we used logistic regression to adjust associations of 3-month mRS and death, institutionalization, and/or post-stroke dementia for age/sex, and estimate age/sex-adjusted probability (“weights”) of these outcomes at 1-year through 5-years for each mRS grade. We did not adjust for potential confounding factors other than age and sex in these regressions, as prior analyses of long-term recovery and cause-specific mortality outcomes in OXVASC showed that outcomes were fairly similar among patients with different levels of stroke severity (per NIHSS), different subtypes, and pre-morbid disease/disability burden, once we stratified by 3-month mRS [27]. We used k-fold cross-validation to generate the AUC (area under the receiver-operating-characteristics curve, a discrimination diagnostic) for each logistic regression model, randomly splitting the dataset into 10 equally sized groups [29]. As a model calibration diagnostic, we used the Hosmer-Lemeshow test [30]. For cost- and QALE-based weights, we stratified mean costs and QALEs by 3-month mRS, deriving 95% confidence-intervals (CIs) from 1000-bootstrap estimates. Analyses were repeated after excluding patients with pre-morbid mRS>2, then pre-morbid mRS>1 to reflect exclusion criteria of many stroke trials. We also stratified results by age (>75 versus <75) and sex. We calculated the 5-year QALE difference between each increment of the mRS for the overall cohort, and compared these values to the estimated minimal clinically important differences (MCIDs) for utility in other diseases, which have been on the order of 0.04-0.10 [31,32]. We examined the influence of recurrent events by recalculating weights for probability of death/dementia/institutionalization after stratifying our data by whether or not the patients had recurrent strokes or other vascular events during 5-year follow-up.

We applied the cohort-derived weights for probability of death/dementia/institutionalization (Pr_DDI_), costs, and QALE to analyses of eight trials of thrombectomy for acute ischaemic stroke (MR CLEAN, ESCAPE, SWIFT-PRIME, EXTEND-IA, REVASCAT, THRACE, DAWN, DEFUSE-3); five were included in a 2016 meta-analysis [4,[33], [34], [35]]. Our analysis used a t-test, as demonstrated for the UW-mRS, [14] wherein we replaced mRS scores for trial patients with corresponding weights (e.g. 5-year Pr_DDI_) and then compared means in treatment and control groups. We assigned mRS=6 a Pr_DDI_=1 for meeting the death endpoint, and cost/QALE weight=0 for costs and QALYs beyond 3-months. We compared our results to conventional ordinal logistic regression and dichotomous/binary logistic regression for “favourable” mRS 0-2 or 0-1. We also compared 1-year and 5-year differences between treatment and control arms in costs, QALE, and Pr_DDI_, estimated with weighted ordinal versus linear ordinal and weighted dichotomous analyses. For the linear ordinal analyses for estimating 1-year and 5-year differences, we used the same weights for mRS=0 and mRS=6 for Pr_DDI_ and QALE, and for mRS=0 and mRS=5 for costs, but then derived the weights for the remaining mRS grades from the slope of the line between the two extreme points. For the weighted dichotomous analyses, we used common Pr_DDI_, cost, and QALE-weights for mRS 0-2 and 3-5, or 0-1 and 2-5, derived as above, weighting mRS=6 the same way.

In light of the publication of 1-year mortality and quality-of-life data from REVASCAT [36] and 2-year data from MR CLEAN,[37] we also applied 1-year and 2-year mortality and QALE weights to analyses of the 3-month outcome data from REVASCAT and MR CLEAN respectively, to compare our estimates of mortality and QALY differences to those actually reported by these two trials.

Significance was set at *p*<0.050.

### 2.2 Role of the funding source

The study funders had no role in study design; data collection, analysis, or interpretation; or the writing of the report. The corresponding author had full access to all data in the study and had final responsibility for the decision to submit for publication.

## 3 Results

Among 1,607 patients with index ischaemic strokes, 181(11.3%) died within 3-months. Of 1,426 3-month survivors, 42(2.9%) were institutionalized pre-stroke, and 144(10.1%) had pre-stroke dementia (baseline characteristics in **Appendix 5**). Complete follow-up data for death, dementia, institutionalization, and costs were available for 1,403(98.4%) survivors. Of 23 excluded survivors, 19 refused follow-up and 4 had mRS assessments only beyond 3-months. 173(12.1%) had not reached 5-year follow-up but had 3-year data. Over 5-years, 465(32.6%) 3-month survivors died, 312(22.3%) of those dementia-free pre-stroke developed dementia, and 229(16.6%) became institutionalized (121[52.8%] had dementia). 652/1,281(50.9%) patients with complete follow-up met endpoints of death, dementia, or institutionalization by 5-years (flow diagram in **Appendix 6**).

Plotting 5-year outcomes against the theoretically-weighted mRS with a step-change at mRS 2-3 resulted in better-fitting linear regressions than the standard mRS (**Appendix 7**), with RMSEs 18-73% lower (**Appendix 8**), demonstrating relative non-linearity of the relationship between 3-month mRS and these outcomes. Using theoretical weights with a step-change at mRS 1-2 or 3-4 resulted in poorer-fitting regressions, identifying mRS 2-3 as the biggest transition in the scale.

**Fig. 1:**
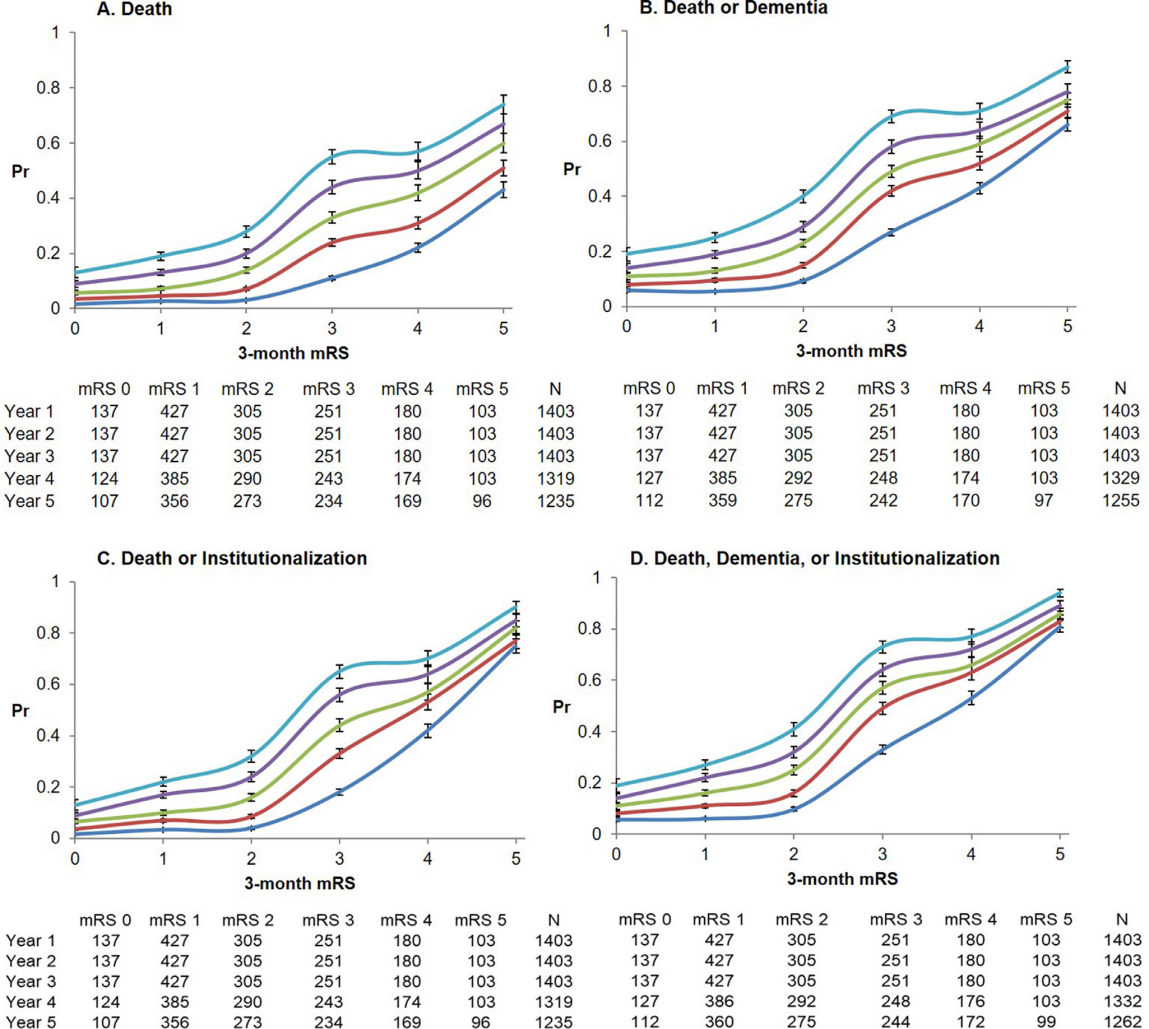
Estimated probability of death, dementia, and/or institutionalization for 1-year to 5-years post-stroke, by 3-month mRS. Age- and sex-adjusted probabilities estimated from logistic regressions for 1-year (dark blue), 2-year (red), 3-year (green), 4-year (purple), and 5-year (light blue) outcomes of (A) death, (B) death or post-stroke dementia, (C) death or post-stroke institutionalization, and (D) death, dementia, or institutionalization, for 3-month survivors of ischaemic stroke, stratified by 3-month mRS (n=1,403). Bars represent 95% confidence intervals. The cumulative number of patients contributing to each estimate are shown, excluding censored cases who had not yet reached that follow-up point.

On performing age/sex-adjusted logistic regression to derive probability-weights for each mRS score, the odds of 5-year death, dementia, and/or institutionalization indeed rose non-linearly with 3-month mRS, with a step-change between mRS 2 and 3 (**Appendix 9**). Plotting probability-weights for each mRS score from these regressions showed an S-shaped curve in each case beyond 1-year (Fig. 1; **Appendix 10**). There was little difference in probability-weights at 1-year between mRS 0, 1, and 2 (e.g. 1-year Pr_DDI_ for mRS 0=0.058, 1=0.061, 2=0.098), with small differences emerging over subsequent years (e.g. 5-year Pr_DDI_ for mRS 0=0.19, 1=0.27, 2=0.41). Additional jumps were seen between mRS 4 and 5 across outcomes. The models used to derive probability-weights all had excellent discrimination with AUC≥0.8 following k-fold cross-validation, and good calibration with non-significant Hosmer-Lemeshow tests (see **Appendix 11–14**). Similar trends were seen upon excluding patients with pre-morbid mRS>2 or >1 (**Appendix 15-16**), or stratifying results by age (**Appendix 17 and 18**) and sex (**Appendix 19 and 20**). Probabilities of attaining endpoints (especially death) were generally lower in younger patients (**Appendix 17**). The probability-weights from models with men and women and those with/without recurrent stroke were similar to those from the overall cohort, generally differing by ≤0.10, but many weights for those aged<75 and >75 fell outside this range (**Appendix 21**).

**Fig. 2:**
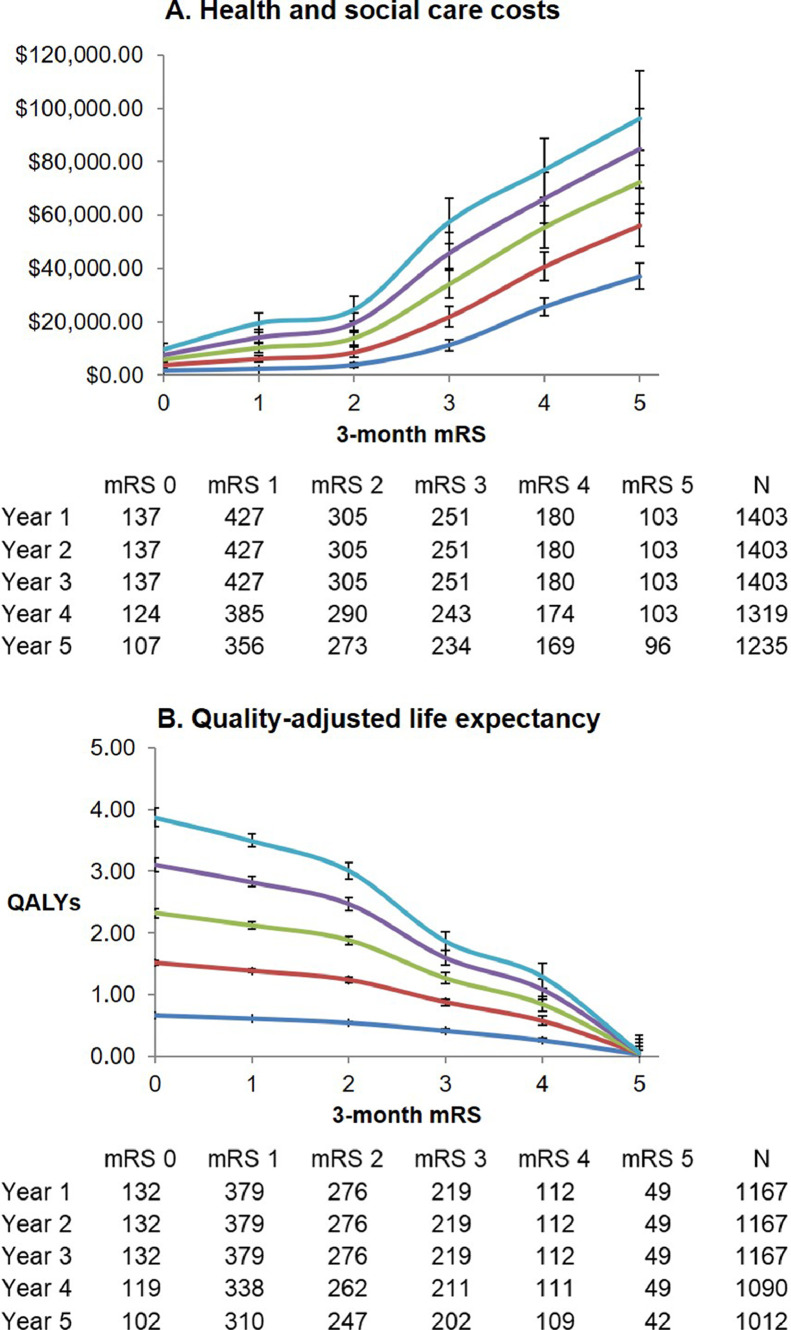
Estimated costs and quality-adjusted life expectancy for 1-year to 5-years post-stroke, by 3-month mRS. Estimated (A) 5-year health and social care costs and (B) quality-adjusted life expectancy (in quality-adjusted life-years, QALYs) for 1-year (dark blue), 2-years (red), 3-years (green), 4-years (purple), and 5-years (light blue) post-stroke for 3-month survivors of ischaemic stroke, stratified by 3-month mRS (n=1,403). Bars represent 95% confidence intervals. The cumulative number of patients contributing to each estimate are shown, excluding censored cases who had not yet reached that follow-up point as well as missing data.

Similar non-linear relationships were observed between 3-month mRS and health/social care costs and QALE over post-stroke years 1 through 5 (Table.1,.**Appendix 22**). Costs generally rose and QALE fell with rising mRS scores (Fig. 2), but again the curves were generally flatter between mRS 0-2 with a step-change at 2-3 (e.g. mean difference in 5-year QALE for mRS 0 versus 1=0.38, 95%CI 0.19-0.57; 1 vs 2=0.48, 0.31-0.66; 2 vs 3=1.15, 0.94-1.33). The lowest mean difference in QALYs at 5-years between two mRS grades in our cohort was 0.38 (mRS=0 vs mRS=1), which was considerably higher than the estimated MCIDs for utility of 0.04-0.10 in other diseases [31,32]. Whereas QALEs showed a greater drop between mRS 4 and 5 than between mRS 3 and 4 (e.g. mean difference in 5-year QALE for mRS 3 vs 4: 0.57, 95%CI 0.31-0.81; 4 vs 5: 1.23, 0.90-1.57), there was essentially a linear trend in costs between mRS 3, 4, and 5 (e.g. mean difference at 5-years for mRS 3 vs 4: $19,476, 95%CI $5,700-33,318; 4 vs 5: $19,272, -$1,141-40,510). Similar results were obtained upon excluding patients with pre-morbid mRS>2 or >1 (**Appendix 23 and 24**). When results were stratified by age (**Appendix 25 and 26**) and sex (**Appendix 27 and 28**), the main difference was that cost curves between mRS 0-2 were flatter and changed little over 5-years for men and those aged<75 (**Appendix.25, 27**). Generally higher healthcare utilization among women resulted in a less pronounced step-change in costs between mRS 2 and 3 in women (e.g. mean difference in 5-year costs for mRS 2 vs 3 in men: $38,462, 95%CI $25,768-51,242; in women: $25,275, $11,314-37,979). 3-month mRS=5 was associated with near-zero QALE at all follow-up years, with mean 5-year QALE<0 (-0.22) in those aged<75. Overall, 5-year QALE “weights” estimated for different groups were similar to those from the whole cohort, generally differing by ≤0.50 (**Appendix 29**).

**Fig. 3:**
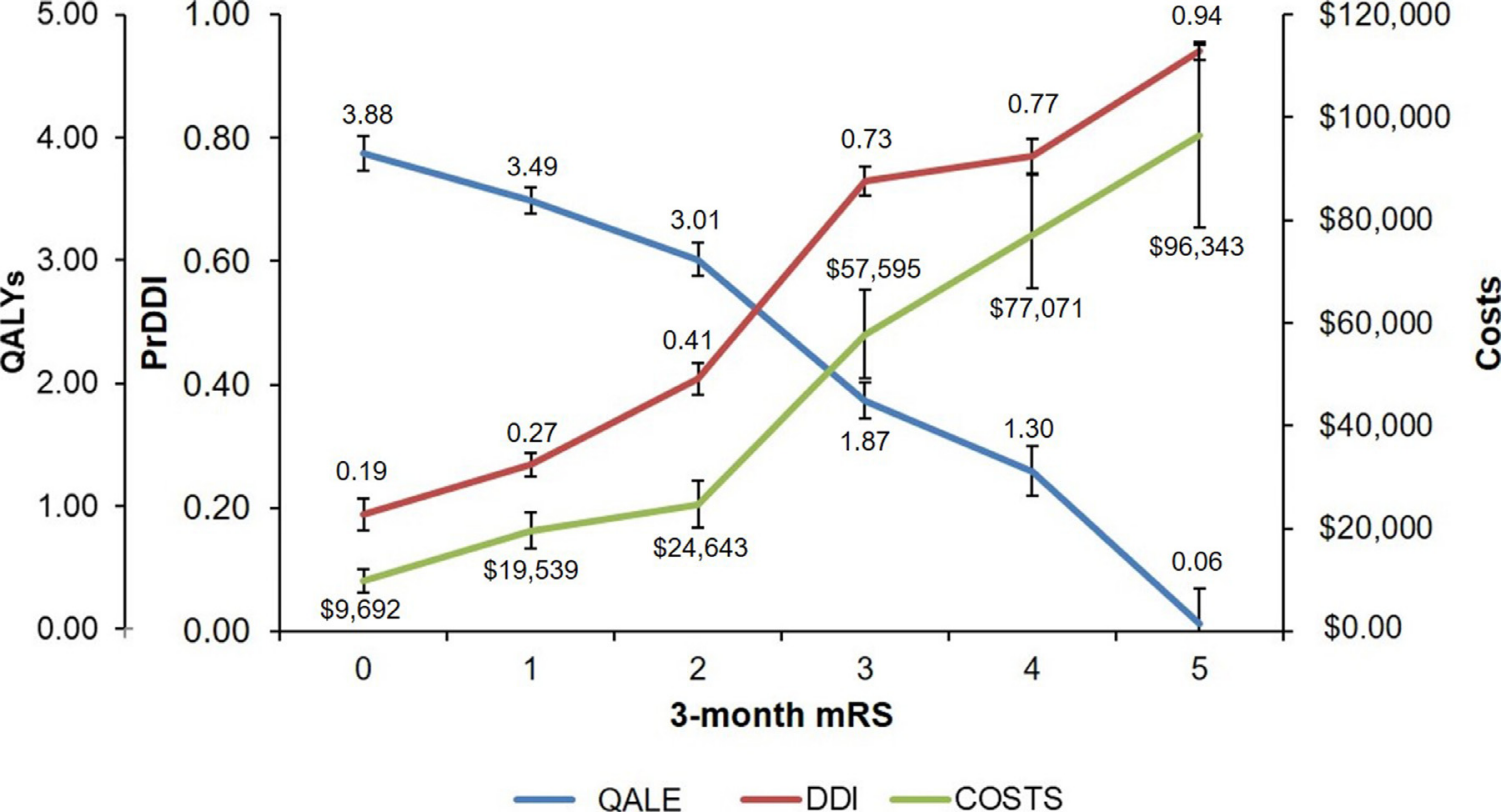
Proposed weights for the mRS based on 5-year post-stroke outcomes. Weights are shown for each 3-month mRS score using the estimated 5-year probability of death, dementia, and/or institutionalization (DDI, red – age/sex-adjusted), 5-year health and social care costs (green), and 5-year quality-adjusted life expectancy (QALE, blue). Each set of weights is plotted on its own y-axis, and the values for the proposed weights (means) are shown adjacent to their respective points on the graph. Bars represent 95% confidence intervals.

Applying 5-year weights for Pr_DDI_, costs, and QALE (Fig. 3) to weighted ordinal analyses of pooled mRS data from recent thrombectomy trials, endovascular therapy is predicted to confer a 11% lower risk (95%CI 9-14%) of death/dementia/institutionalization, a $10,193(7,405-12,981) reduction in health/social-care costs, and add 0.55(0.43-0.66) QALYs over 5-years versus control treatments. Estimated treatment effects at 1-year and 5-years were consistently higher using the weighted ordinal approach compared to linear ordinal or weighted dichotomous approaches; for example, 5-year estimated cost differences with the ordinal approach were about 21% higher than with a linear ordinal approach, 29% higher than with the 0-2/3-6 dichotomy, and over three-fold higher than with the 0-1/2-6 dichotomy (Table 2). Similar differences were seen for estimated 5-year treatment effects for the individual trials (**Appendix 30–33**), with many of the effects also becoming insignificant with a 0-1/2-6 dichotomy. Similar P-values were generally obtained with the weighted ordinal approach as from conventional ordinal analysis, with the notable exception of REVASCAT, which was non-significant using unadjusted conventional ordinal or linear ordinal analysis (**Appendix 32**) but significant using weighted mRS approaches (**Appendix 33**).

On applying 1-year mortality (**Appendix 11**) and QALE weights (Table 1) to 3-month mRS data for REVASCAT, we estimated a 2.5% lower mortality(95%CI -7.1% to 12.0%, p=0.61) and 0.06 additional QALY(0.003-0.13, *p*=0.03) in the treatment arm at 1-year. This was comparable to the non-significant 1% mortality difference and 0.12(0.03-0.22, *p*=0.01) utility difference reported at 1-year in REVASCAT.[36] On applying 2-year mortality and QALE weights to the 3-month mRS data for MR CLEAN, we estimated a 5.5% lower mortality(-0.5% to 11.4%, *p*=0.07) and 0.14 additional QALY(0.06-0.23, p=0.001) in the treatment arm: very similar to the 5% mortality (*p*=0.46) and 0.10 utility difference (0.03-0.16, p=0.006) reported at 2-years in MR CLEAN [37].

**Table 1:** Estimated quality-adjusted life expectancy (QALE) at 1-year, 2-years, 3-years, 4-years, and 5-years post-stroke for each 3-month mRS score in 3-month ischaemic stroke survivors.

3-month	Year 1	Year 2	Year 3	Year 4	Year 5
mRS	QALYS (95%CI)	QALYS (95%CI)	QALYS (95%CI)	QALYS (95%CI)	QALYs (95%CI)*
**0**	0.67 (0.65-0.68)	1.52 (1.48-1.56)	2.32 (2.25-2.40)	3.11 (3.00-3.22)	3.88 (3.73-4.03)
**1**	0.62 (0.60-0.63)	1.39 (1.36-1.42)	2.12 (2.07-2.18)	2.83 (2.75-2.91)	3.49 (3.39-3.61)
**2**	0.55 (0.53-0.56)	1.24 (1.20-1.28)	1.89 (1.81-1.95)	2.48 (2.37-2.57)	3.01 (2.87-3.14)
**3**	0.42 (0.39-0.44)	0.88 (0.83-0.93)	1.27 (1.19-1.36)	1.60 (1.48-1.72)	1.87 (1.72-2.02)
**4**	0.26 (0.23-0.30)	0.58 (0.51-0.66)	0.85 (0.74-0.97)	1.09 (0.93-1.25)	1.30 (1.10-1.51)
**5**	0.042 (-0.0060-0.099)	0.055 (-0.039-0.16)	0.058 (-0.091-0.22)	0.061 (-0.15-0.29)	0.063 (-0.20-0.34)
**N**	1167	1167	1167	1090	1012

Mean difference in QALYs at 5 years for mRS 0 vs 1: 0.38 (95%CI 0.19-0.57), mRS 1 vs 2: 0.48 (0.31-0.66), mRS 2 vs 3: 1.15 (0.94-1.33), mRS 3 vs 4: 0.57 (0.31-0.81), mRS 4 vs 5: 1.23 (0.90-1.57).

EQ-5D data were missing for 236(16.5%) 3-month survivors, due to patients refusing follow-up (n=18) or EQ-5D assessments (n=33), being too ill/disabled (n=89), or not attending follow-up appointments (n=96).

**Table 2:** Effect sizes estimated using 1-year and 5-year probability-, cost-, and QALE-weights for pooled analysis of the modified Rankin Scale (mRS) in recent thrombectomy trials, with a 0-1/2-5 dichotomy, a 0-2/3-5 dichotomy, a linear ordinal approach (assuming equal differences for each state-transition), and a weighted ordinal approach.

Analysis approach	PrDDI Difference at 1-year	PrDDI Difference at 5-years	Cost Difference at 1-year	Cost Difference at 5-years	QALE Difference at 1-year	QALE Difference at 5-years
**EARLY-WINDOW THROMBECTOMY TRIALS**						
Dichotomized 0-1 vs 2-5	**0.06** (0.03-0.08)	**0.06** (0.04-0.08)	**$,1,100** (469-1,730)	**$2,951** (800-5,103)	**0.04** (0.02-0.06)	**0.25** (0.14-0.35)
Dichotomized 0-2 vs 3-5	**0.09** (0.06-0.12)	**0.09** (0.06-0.11)	**$2,547** (1,641-3,453)	**$6,808** (4,012-9,603)	**0.06** (0.04-0.08)	**0.37** (0.26-0.49)
Linear ordinal mRS	**0.10** (0.07-0.13)	**0.09** (0.06-0.11)	**$3,152** (1,983-4,321)	**$7,549** (4,555-10,544)	**0.07** (0.05-0.09)	**0.41** (0.30-0.53)
Weighted ordinal mRS	**0.11** (0.08-0.14)	**0.10** (0.07-0.13)	**$3,889** (2,738-5,041)	**$8,967** (5,904-12,029)	**0.08** (0.06-0.10)	**0.48** (0.36-0.61)
**LATE-WINDOW THROMBECTOMY TRIALS**						
Dichotomized 0-1 vs 2-5	**0.09** (0.03-0.15)	**0.09** (0.05-0.14)	**$1,592** (260-2,923)	**$4,198** (-422-8,819)	**0.06** (0.02-0.10)	**0.38** (0.16-0.61)
Dichotomized 0-2 vs 3-5	**0.16** (0.10-0.22)	**0.16** (0.11-0.21)	**$4,769** (2,886-6,653)	**$12,823** (6,872-18,774)	**0.11** (0.07-0.15)	**0.68** (0.45-0.91)
Linear ordinal mRS	**0.16** (0.11-0.22)	**0.14** (0.09-0.19)	**$5,135** (2,449-7,711)	**$12,291** (5,661-18,922)	**0.12** (0.08-0.16)	**0.67** (0.44-0.91)
Weighted ordinal mRS	**0.19** (0.13-0.26)	**0.17** (0.12-0.22)	**$6,815** (4,292-9,337)	**$15,667** (8,973-22,360)	**0.14** (0.09-0.18)	**0.83** (0.57-1.09)
**ALL THROMBECTOMY TRIALS**						
Dichotomized 0-1 vs 2-5	**0.06** (0.04-0.09)	**0.07** (0.05-0.09)	**$1,193** (624-1,763)	**$3,192** (1,243-5,141)	**0.04** (0.03-0.06)	**0.27** (0.18-0.37)
Dichotomized 0-2 vs 3-5	**0.10** (0.07-0.13)	**0.10** (0.08-0.12)	**$2,956** (2,139-3,772)	**$7,916** (5,385-10,446)	**0.07** (0.05-0.08)	**0.43** (0.33-0.53)
Linear ordinal mRS	**0.11** (0.09-0.14)	**0.09** (0.07-0.12)	**$3,516** (2,450-4,581)	**$8,420** (5,689-11,151)	**0.08** (0.06-0.10)	0.46 (0.36-0.56)
Weighted ordinal mRS	**0.13** (0.10-0.15)	**0.11** (0.09-0.14)	**$4,422** (3,373-5,472)	**$10,193** (7,405-12,981)	**0.09** (0.07-0.11)	**0.55** (0.43-0.66)

All positive values indicate a favourable difference between treatment and control arms (i.e control minus treatment for probability and cost, treatment minus control for QALE). Values are presented as means with 95% confidence intervals. PrDDI = probability of death, dementia, or institutionalization. QALE = Quality-adjusted life expectancy.

## 4 Discussion

In this population-based prospective cohort of ischaemic stroke survivors, we demonstrated non-linear relationships between the 3-month mRS and 5-year outcomes of death, dementia, institutionalization, costs, and QALE, finding that a theoretical set of mRS weights with a step-change at mRS 2 to 3 linearized the data better than standard mRS values. We then derived probability weights for each mRS grade for death, dementia, and/or institutionalization at 1-year through 5-years, and estimated health/social-care costs and QALE over this period for each mRS grade. Similar trends were seen upon excluding patients with pre-morbid disability and stratifying by age and sex. In addition to providing normative data for prognostication and cost-effectiveness analyses, these findings have implications for analysis and interpretation of trials using the mRS.

Firstly, the incremental rise in odds of death/dementia/institutionalization, and incremental fall in QALE (without overlapping confidence intervals) with each step up the mRS, further establish the predictive validity of the mRS in ischaemic stroke,[3] and imply that any shifts in 3-month mRS towards lower grades are likely to be clinically meaningful and valued by patients. The lowest mean difference in QALYs at 5-years between two consecutive mRS grades in our cohort was 0.38 (mRS=0 vs mRS=1), higher than reported MCIDs of 0.04-0.10 for utility in other conditions, further highlighting the importance of using ordinal rather than dichotomous analyses of the mRS in trials.

Secondly, the demonstrated unequal (non-linear) differences in outcomes between mRS grades underscore the need to weight the mRS in ordinal analyses, building upon prior work by groups like the OAST (Optimising Analysis of Stroke Trials) Collaboration that demonstrated the superiority of ordinal over dichotomous analyses [5]. Different state-transitions clearly differ in prognosis, quality-of-life, and associated costs. By acknowledging that certain transitions are more significant whilst retaining the full range of the scale, this approach facilitates analyses that identify clinically meaningful – not just statistically significant – treatment differences.

Thirdly, building upon the UW-mRS – recently used in the DAWN trial [34] – the estimated probabilities (5-year death/dementia/institutionalization), costs, and QALEs in this paper have the added advantage of providing weights that reflect the long-term trajectories of these patients and the burden of their disease, versus a cross-sectional quality-of-life rating. Similarly, our combination of mortality and quality-of-life data into mRS-stratified QALEs also expands upon the mRS-stratified health utilities (sans mortality) published in a recent analysis of the Virtual International Stroke Trials Archive (VISTA),[38] which drew on a much larger sample of 3,858 patients and had the added strength of accounting for country-specific differences by using value sets from 13 countries. Moreover, the shape of the UW-mRS curve differs from that observed for the long-term outcomes in our cohort, including the QALE curves; in particular, the greatest utility value steps on the UW-mRS are between mRS 3, 4, and 5 and there is no step-change between mRS 2 to 3 [14]. As we have shown using the example of thrombectomy, the mean difference between treatment/control groups provided through this t-test approach can provide a meaningful “effect size” that reflects the predicted long-term difference derived from the treatment (e.g. predicted lowering of probability of 5-year death/dementia/institutionalization or 5-year costs, or increase in 5-year QALE). Such results offer greater immediate interpretability than conventional ordinal mRS, expressing treatment differences in terms typically used in population-level healthcare decisions. Furthermore, as we have shown, obtaining such estimates using a weighted ordinal approach better demonstrates the expected long-term persistence and/or magnification of treatment effects of therapies like thrombectomy than dichotomous or linear approaches. As an important initial validation of our weights, our estimates of 1-year and 2-year mortality and utility differences were quite comparable to those actually observed in REVASCAT and MR CLEAN [36,37]. We used a t-test for our illustrative trial analyses owing to its simplicity; however, in practice, using a different approach like a generalized linear model after substituting weights for the mRS would give similarly interpretable effect sizes whilst also allowing adjustment for other baseline variables.

Despite the aforementioned advantages, weighted ordinal approaches to analyzing the mRS may not necessarily be statistically superior to unweighted ordinal approaches [39]. For example, the UW-mRS was not superior to unweighted ordinal analyses when applied to a sample of prior stroke trials,[14] and a recent simulation study found that the UW-mRS was less statistically efficient than the unweighted ordinal approach (power 85% versus 87%) [40]. That being said, the UW-mRS has salient differences from our prognosis-based weights proposed in this paper – chief of which is that it uses a cross-sectional assessment of utility as compared to the 5-year follow-up data used for our estimates – so results may differ. For instance, within the very limited sample of thrombectomy studies that we examined in this paper, we found that the REVASCAT trial was non-significant using unadjusted conventional or unweighted ordinal analysis but was significant using our weighted ordinal approaches (the adjusted analysis was positive, as published). Nevertheless, future studies should compare how these different weighted approaches perform across a wider range of real or simulated trial data, including potential differences in statistical power.

Besides, the question of which weights should be favoured for primary outcome analysis of the mRS is more complicated. Since the mRS was not designed as a measure of cognitive disability or health-related quality-of-life, it may be argued that there is a risk of over-fitting or changing the nature of the mRS by weighting it using such outcomes as opposed to simpler measures like mortality. In this regard, it is perhaps reassuring that we found a similar non-linear (S-shaped) relationship between the 3-month mRS and 5-year outcomes regardless of whether we examined QALE, dementia, death, or institutionalization. Cost-based weights reflect disease burden to both the patient and health system, but risk rewarding deaths (mRS=6) and will vary by age, sex (as in our cohort), and healthcare system. Using probability weights like those for death/dementia/institutionalization avoids the issue of weighting mRS=6, but these weights also varied considerably by age. However, 5-year QALE weights were relatively consistent across different age groups. In this regard, QALE weights seem most appealing, and by combining mortality and quality-of-life data, they can inform treatment evaluations and resource allocation. The estimate of treatment effect using such weights would be a single easily understood value for the difference in QALE between the two trial strategies. An important limitation of the UW-mRS, as discussed in recent publications, is the high variability in utility values within each mRS category and across time post-stroke [40,41]. By aggregating utility assessments from multiple time-points up to 5-year follow-up from a population-based cohort, our QALE weights may mitigate this limitation by accounting for some of the utility variability over time.Even if the QALE (or other) weights are used for secondary or tertiary analyses in stroke trials, they could add to the meaningful interpretation of the trial results, including in otherwise neutral trials, where they may suggest potential treatment effects worthy of further study.

Our analysis has several strengths, including a robust population-based design with high ascertainment of incident strokes; completeness of follow-up for disability, dementia, death, institutionalization, and cost data; and replication of findings for various subgroups. However, there are potential limitations. Firstly, the generalizability of our mRS weights may be limited by differences in practice settings, case-mix, ethnicity, or cultural values. The OXVASC population is from a geographically distinct part of the United Kingdom, is 95% White, relatively elderly (mean age 73-years), and is cared for within a publicly funded healthcare system with relatively accessible acute and post-acute stroke care. Mortality and morbidity-based weights may differ considerably in lower and middle-income countries. Some of the patients in our cohort had their index stroke up to 18 years ago, during which time stroke care and outcomes have changed considerably. As such, our weighted mRS may be seen as a work in development that requires further validation and refinement using stroke datasets from different populations like the Northern Manhattan Study [42] or the Clinical Research Collaboration for Stroke in Korea [43]. Secondly, the weights generated for 3-month mRS may not be generalizable to mRS measurements at other time-points. Thirdly, if a new therapy modifies risks of long-term dementia, institutionalization, quality-of-life, or mortality beyond reduction in stroke-related disability, then our probability-weighted mRS approach could under-estimate that benefit. Our estimates of resource use may also not be representative in such cases. However, this would still be an improvement over conventional approaches that do not capture prognostic implications, and could serve as a useful “shorthand” estimate of cost-effectiveness. Fourthly, further data is required to demonstrate the clinical interpretability of our weighted mRS data in practice; for example, this could include focus groups with clinicians and trialists who are shown trial data using different ordinal approaches and asked to choose which presentation is easiest to understand. Indeed, weighting the mRS by different prognoses or costs is not the only method to potentially improve the clinical interpretability of mRS data in clinical trials. Work by other groups may examine combining other scales with the mRS to create a single, aggregate measure, or using other scales like the Barthel Index [44] or Functional Independence Measure [2] to add granularity, particularly with more intermediate mRS scores. It could be argued that a weighted approach may be more useful for more granular scales, but striking the right balance between granularity and a manageable number of state-transitions for weighting can be complicated; the 20-point Barthel Index, for example, may need up to 21 weights. Fifthly, whilst we included the costs of long-term care in addition to hospital- or clinic-based costs, we did not include important indirect costs like social services assistance and productivity losses that constitute a major proportion of longer-term post-stroke costs [45]. Including such costs in cost-based weights would likely further amplify differences between higher and lower mRS scores. Sixthly, our weights are limited to patients with ischaemic stroke; separate weights for the mRS would ideally need to be derived for patients with haemorrhagic stroke or other conditions, in whom mRS-stratified outcomes may be considerably different. We also did not adjust for all potential confounding factors that may influence long-term outcomes; future work may seek to derive separate weights for patients in different stroke subtypes, for example. We partially accounted for the influence of comorbidities in the current paper by examining the weights for different outcomes not only in the overall cohort but also after removing patients with higher levels of pre-morbid mRS, which captures pre-stroke comorbidity burden reasonably well [46].

In conclusion, cohort-derived data for clinical endpoints or health-economic outcomes of interest can be used to inform prognostication, cost-effectiveness, and weighted ordinal analyses of the mRS in clinical trials. Analyses of other stroke cohorts could validate our findings. Cohort studies of other neurological conditions may derive similar weights for commonly-used ordinal scales to examine their predictive validity and facilitate clinically-meaningful ordinal analyses.

## Contributors

AG collected data, performed statistical analysis and interpretation, and wrote and revised the manuscript. RLF and STP acquired data and contributed to statistical analysis. PMR conceived and designed the study, provided study supervision and funding, and wrote and revised the manuscript.

## Funding/Support

Wellcome Trust, Wolfson Foundation, NIHR Oxford Biomedical Research Centre, Rhodes Trust.

## Declaration of Competing Interest

Dr. Ganesh reports membership in the editorial boards of Neurology, Neurology: Clinical Practice, and Stroke; speaker honoraria from NHS Health Education England and The Meritas Seminar Series, Oxford; consulting fees from MD Analytics, MyMedicalPanel, Adkins Research Group, and Genome BC; research support from The Rhodes Trust and Wellcome Trust; and stock/stock options from SnapDx, TheRounds.ca, and Advanced Health Analytics (AHA Health Ltd). Dr. Luengo-Fernandez has nothing to disclose. Dr. Pendlebury has nothing to disclose. Prof. Rothwell has nothing to disclose.

## Appendix Supplementary materials

Image, application 1

Image, application 2
