# Supplementary material for: Weights for ordinal analyses of the modified Rankin Scale in stroke trials: A population-based cohort study
Source: eClinicalMedicine. 2020 Jun 15;23:100415. doi: 10.1016/j.eclinm.2020.100415 (PMC7300241; doi:10.1016/j.eclinm.2020.100415)
Supplement: Supplementary file 1 [file mmc1.docx]

**Web Appendix**

| **Web appendix** | **Title** | **Page** |
| --- | --- | --- |
| 1 | Categories of the modified Rankin Scale (mRS) | 3 |
| 2 | Medical conditions for which the mRS (or related Rankin Scale or Oxford Handicap Scale) has been used as an outcome measure, and their publication in leading journals since 2000 | 4 |
| 3 | Supplementary Methods **–** Ascertainment of Dementia in OXVASC | 5 |
| 4 | Theoretical examples of non-linear weights for the modified Rankin Scale (mRS), derived from linear spline with knots at (A) 1 and 2, (B) 2 and 3, or (C) 3 and 4, and corresponding coefficients of 1, 4, and 2 for the resulting three segments. | 7 |
| 5 | Patient sample and characteristics for 3-month survivors of ischaemic stroke (n=1,426) | 8 |
| 6 | Flow diagram for patients with ischaemic stroke occurring from April 2002 to March 2014 in the Oxford Vascular Study, followed until 15-May-2017 | 9 |
| 7 | Representative plots of 5-year outcomes – proportion dead (A-B) and health/social care costs (C-D) – against the 3-month mRS using its standard values (A,D) versus using the theoretical weights shown in eFigure 1B. | 10 |
| 8 | Root mean squared error (RMSE) for linear regression of various 5-year outcomes (dependent variable) on the 3-month mRS (independent variable) in 3-month survivors of ischaemic stroke, with the mRS plotted as a linear scale (0, 1, 2, 3, 4, 5) versus also incorporating a spline (and thus a weighted step-change) at transitions 1-2, or 2-3, or 3-4. | 11 |
| 9 | Odds and probability-weights for 5-year death, dementia, or institutionalization for each 3-month mRS score (age/sex-adjusted) in 3-month ischaemic stroke survivors with full 5-years of follow-up, or who met the endpoint of interest between 3-months and 5-years | 12 |
| 10 | Age- and sex-adjusted probabilities estimated from logistic regressions for 1-year (dark blue), 2-year (red), 3-year (green), 4-year (purple), and 5-year (light blue) outcomes of (A) post-stroke institutionalization and (B) post-stroke dementia for 3-month survivors of ischaemic stroke (n=1,425), stratified by 3-month mRS. Bars represent 95% confidence intervals. | 13 |
| 11 | Age- and sex-adjusted probabilities of death in Years 1 through 5, estimated from logistic regressions in 3-month survivors of ischaemic stroke, grouped by 3-month mRS. | 14 |
| 12 | Age- and sex-adjusted probabilities of death or post-stroke dementia in Years 1 through 5, estimated from logistic regressions in 3-month survivors of ischaemic stroke, grouped by 3-month mRS. | 15 |
| 13 | Age-/sex-adjusted probabilities of death or post-stroke institutionalization in Years 1 to 5, estimated from logistic regressions in 3-month survivors of ischaemic stroke, grouped by 3-month mRS | 16 |
| 14 | Age- and sex-adjusted probabilities of death, post-stroke dementia, or institutionalization in Years 1 through 5, estimated from logistic regressions in 3-month survivors of ischaemic stroke, grouped by 3-month mRS | 17 |
| 15 | Age- and sex-adjusted probabilities estimated from logistic regressions for 1-year (dark blue), 2-year (red), 3-year (green), 4-year (purple), and 5-year (light blue) outcomes of (A) death, (B) death or post-stroke dementia, (C) death or post-stroke institutionalization, and (D) death, dementia, or institutionalization for 3-month survivors of ischaemic stroke, stratified by 3-month mRS, excluding patients with pre-morbid mRS>2 (n=1,171). | 18 |
| 16 | Age- and sex-adjusted probabilities estimated from logistic regressions for 1-year (dark blue), 2-year (red), 3-year (green), 4-year (purple), and 5-year (light blue) outcomes of (A) death, (B) death or post-stroke dementia, (C) death or post-stroke institutionalization, and (D) death, dementia, or institutionalization for 3-month survivors of ischaemic stroke, stratified by 3-month mRS, excluding patients with pre-morbid mRS>1 (n=984). | 19 |
| 17 | Unadjusted probabilities estimated from logistic regressions for 1-year (dark blue), 2-year (red), 3-year (green), 4-year (purple), and 5-year (light blue) outcomes of (A) death, (B) death or post-stroke dementia, (C) death or post-stroke institutionalization, and (D) death, dementia, or institutionalization for 3-month survivors of ischaemic stroke, stratified by 3-month mRS, restricted to those aged <75 years (n=669). | 20 |
| 18 | Unadjusted probabilities estimated from logistic regressions for 1-year (dark blue), 2-year (red), 3-year (green), 4-year (purple), and 5-year (light blue) outcomes of (A) death, (B) death or post-stroke dementia, (C) death or post-stroke institutionalization, and (D) death, dementia, or institutionalization for 3-month survivors of ischaemic stroke, stratified by 3-month mRS, restricted to those aged >75 years (n=733). | 21 |
| 19 | Unadjusted probabilities estimated from logistic regressions for 1-year (dark blue), 2-year (red), 3-year (green), 4-year (purple), and 5-year (light blue) outcomes of (A) death, (B) death or post-stroke dementia, (C) death or post-stroke institutionalization, and (D) death, dementia, or institutionalization for 3-month survivors of ischaemic stroke, stratified by 3-month mRS, restricted to male sex (n=748). | 22 |
| 20 | Unadjusted probabilities estimated from logistic regressions for 1-year (dark blue), 2-year (red), 3-year (green), 4-year (purple), and 5-year (light blue) outcomes of (A) death, (B) death or post-stroke dementia, (C) death or post-stroke institutionalization, and (D) death, dementia, or institutionalization for 3-month survivors of ischaemic stroke, stratified by 3-month mRS, restricted to female sex (n=655). | 23 |
| 21 | Comparison of probability weights for 5-year death, dementia, or post-stroke institutionalization, derived from logistic regressions in 3-month survivors of ischaemic stroke and grouped by 3-month mRS, in: all patients, excluding premorbid mRS>2, excluding premorbid mRS>1, age<75, age>75, men, and women | 24 |
| 22 | Estimated total health and social care costs at 1-year, 2-years, 3-years, 4-years, and 5-years post-stroke for each 3-month mRS score in 3-month ischaemic stroke survivors. | 25 |
| 23 | Estimated (A) 5-year health and social care costs and (B) quality-adjusted life expectancy (in quality-adjusted life-years, QALYs) for 1-year (dark blue), 2-years (red), 3-years (green), 4-years (purple), and 5-years (light blue) post-stroke for 3-month survivors of ischaemic stroke, stratified by 3-month mRS, excluding those with pre-morbid mRS>2 (n=1,171). | 26 |
| 24 | Estimated (A) 5-year health and social care costs and (B) quality-adjusted life expectancy (in quality-adjusted life-years, QALYs) for 1-year (dark blue), 2-years (red), 3-years (green), 4-years (purple), and 5-years (light blue) post-stroke for 3-month survivors of ischaemic stroke, stratified by 3-month mRS, excluding those with pre-morbid mRS>1 (n=984). | 27 |
| 25 | Estimated (A) 5-year health and social care costs and (B) quality-adjusted life expectancy (in quality-adjusted life-years, QALYs) for 1-year (dark blue), 2-years (red), 3-years (green), 4-years (purple), and 5-years (light blue) post-stroke for 3-month survivors of ischaemic stroke, stratified by 3-month mRS, restricted to those aged <75 years (n=669). | 28 |
| 26 | Estimated (A) 5-year health and social care costs and (B) quality-adjusted life expectancy (in quality-adjusted life-years, QALYs) for 1-year (dark blue), 2-years (red), 3-years (green), 4-years (purple), and 5-years (light blue) post-stroke for 3-month survivors of ischaemic stroke, stratified by 3-month mRS, restricted to those aged >75 years (n=733). | 29 |
| 27 | Estimated (A) 5-year health and social care costs and (B) quality-adjusted life expectancy (in quality-adjusted life-years, QALYs) for 1-year (dark blue), 2-years (red), 3-years (green), 4-years (purple), and 5-years (light blue) post-stroke for 3-month survivors of ischaemic stroke, stratified by 3-month mRS, restricted to male sex (n=748). | 30 |
| 28 | Estimated (A) 5-year health and social care costs and (B) quality-adjusted life expectancy (in quality-adjusted life-years, QALYs) for 1-year (dark blue), 2-years (red), 3-years (green), 4-years (purple), and 5-years (light blue) post-stroke for 3-month survivors of ischaemic stroke, stratified by 3-month mRS, restricted to female sex (n=655). | 31 |
| 29 | Comparison of 5-year quality-adjusted life expectancies (QALE weights) for 3-month survivors of ischaemic stroke, grouped by 3-month mRS, in: all patients, excluding premorbid mRS>2, excluding premorbid mRS>1, age<75, age>75, men, and women. | 32 |
| 30 | Effect sizes estimated using 5-year probability-, cost-, and QALE-weights for dichotomous analysis of the mRS in recent thrombectomy trials, with a 0-1/2-5 dichotomy, compared to the odds ratio (OR) from binary logistic regression. | 33 |
| 31 | Effect sizes estimated using 5-year probability-, cost-, and QALE-weights for dichotomous analysis of the mRS in recent thrombectomy trials, with a 0-2/3-5 dichotomy, compared to the odds ratio (OR) from binary logistic regression | 34 |
| 32 | Effect sizes for 5-year probability of death/dementia/institutionalization, costs, and QALE, estimated using a “linear” ordinal analysis of the mRS in recent thrombectomy trials, compared to the common odds ratio (cOR) from ordinal logistic regression. | 35 |
| 33 | Effect sizes estimated using a weighted ordinal analysis with 5-year probability-, cost-, and QALE-weights for the mRS in recent thrombectomy trials, compared to the common odds ratio (cOR) from ordinal logistic regression | 36 |
|  |  |  |

**Web Appendix 1. Categories of the modified Rankin Scale (mRS)**[**^15^**](#_ENREF_15)

| **mRS score/category** | **Description** |
| --- | --- |
| **0** | No symptoms at all |
| **1** | Able to carry out all usual duties and activities, despite symptoms |
| **2** | Unable to carry out all previous activities, but able to look after own affairs without assistance |
| **3** | Requiring some help, but able to walk without assistance |
| **4** | Unable to walk without assistance and unable to attend to own bodily needs without assistance |
| **5** | Bedridden, incontinent, and requiring constant nursing care and attention |
| **6** | Dead |

**Web Appendix 2. Medical conditions for which the modified Rankin Scale (or related Rankin Scale or Oxford Handicap Scale) has been used as an outcome measure, and their publication in leading journals since 2000.***

| **Medical condition** | **Estimated publications** | **JAMA group (214)** | **Lancet group (174)** | **NEJM**  **(87 articles)** |
| --- | --- | --- | --- | --- |
| **Cerebrovascular disorders**  Stroke, aneurysms, arteriovenous malformations, cavernomas | 3,770 | X | X | X |
| **Cardiovascular disorders** |  |  |  |  |
| Atrial Fibrillation | 1,030 |  | X | X |
| Cardiac arrest | 202 | X | X | X |
| Coronary artery bypass (or related surgery) | 212 |  | X | X |
| Heart failure or valvular heart disease (including procedures for aortic stenosis, mitral regurgitation) | 201 | X |  | X |
| **Musculoskeletal disorders** |  |  |  |  |
| Low back pain | 171 |  |  |  |
| Osteoarthritis, Rheumatoid arthritis | 118 |  |  |  |
| Myopathies: Dermatomyositis, Polymyositis, Mitochondrial, spinal/bulbar muscular atrophy, muscular dystrophy, anti-SRP, hyper-CK-emia, anti-synthetase syndrome | 105 | X |  |  |
| **Infectious diseases** |  |  |  |  |
| Infective endocarditis | 108 |  |  |  |
| HIV/AIDS | 99 |  |  |  |
| **Non-stroke CNS disorders** |  |  |  |  |
| Traumatic and other acute brain injury | 443 | X |  |  |
| Multiple Sclerosis, ADEM, Neuromyelitis Optica | 336 |  |  |  |
| Ataxias (FXTAS, autoimmune, etc) | 316 |  |  |  |
| Parkinson’s Disease | 307 | X | X |  |
| Alzheimer’s Disease | 294 |  |  |  |
| Epilepsy, PNES, or Conversion Disorder | 269 | X |  |  |
| Subdural hematoma | 242 |  | X |  |
| Cerebral venous sinus thrombosis | 144 |  |  |  |
| Infectious meningitis, encephalitis, or encephalomyelitis | 128 | X | X | X |
| Coma or herniation | 113 |  |  |  |
| Autoimmune encephalitis: anti-NMDA, anti-GAD, anti-LG1, etc | 112 | X | X |  |
| Myelopathies: tethered cord, AVFs, epidural hematoma, etc. | 65 |  |  |  |
| Gliomas | 54 |  |  |  |
| Huntington’s Disease | 37 |  |  |  |
| Normal Pressure Hydrocephalus | 35 |  |  |  |
| Migraine, tension headaches | 35 |  |  |  |
| Progressive Multifocal Leukoencephalopathy | 15 |  |  |  |
| Prion Disease | 15 |  | X |  |
| **Peripheral Nervous System (or Mixed)** |  |  |  |  |
| Polyneuropathies: GBS, CIDP, CMT, paraproteinemic, cryoglobulinemic, vasculitis, Sjogren’s, paraneoplastic, multifocal motor neuropathy, alcohol/thiamine-deficiency | 236 |  | X | X |
| Amyotrophic Lateral Sclerosis | 189 |  |  |  |
| Myasthenia Gravis | 45 |  |  |  |
| **Other general medical or systemic conditions** |  |  |  |  |
| Asthma, COPD | 126 |  |  |  |
| Small-cell lung cancer | 79 |  |  |  |
| Lupus erythematosus | 69 |  |  |  |
| Churg-Strauss syndrome | 17 |  |  |  |
| Ehlers-Danlos syndrome | 16 |  |  |  |

*Each medical condition using/influenced by the mRS was identified by reviewing the titles/abstracts of the first 100 pages of Google Scholar citation results for the two top-cited publications on the Rankin Scale (van Swieten et al 1988, Rankin 1957). Then, the number of publications for each identified disease was estimated using the “search within citing articles” function in Google Scholar for these two publications. There is likely to be some overlap among the publications listed in different categories (e.g. owing to papers that include patients with both rheumatoid arthritis and low back pain).

**Web Appendix 3. Supplementary Methods – Ascertainment of dementia in OXVASC**

Cognitive testing was done at all follow-ups using ≥1 of the Mini-Mental State Examination (MMSE),[^1^](#_ENREF_1) Montreal Cognitive Assessment (MoCA),[^2^](#_ENREF_2) or Telephone Interview for Cognitive Status-modified (TICSm),[^3^](#_ENREF_3) all of which have been validated against the National Institute of Neurological Disorders and Stroke-Canadian Stroke Network (NINDS-CSN) Vascular Cognitive Impairment Harmonization Standards Neuropsychological Battery.[^4-7^](#_ENREF_4) The MMSE was done at all time points until April 1, 2005, when the baseline MMSE was replaced by the 10 point Abbreviated Mental Test Score (AMTS).[^8^](#_ENREF_8)^,^ [^9^](#_ENREF_9) From April 2007, the MoCA was introduced for the 6-month, 1-year, and 5-year follow-ups as recommended by the NINDS-CSN Vascular Cognitive Impairment Harmonization Standards Working Group.[^4^](#_ENREF_4) The TICSm or telephone MoCA (out of 12)[^6^](#_ENREF_6) was done by telephone when face-to-face follow-up was not feasible. The Informant Questionnaire for Cognitive Decline in the Elderly (IQCODE) was also administered to an informant whenever possible to further assess pre-morbid cognitive functioning.[^10^](#_ENREF_10)

Dementia was defined as pre- or post-stroke. Pre-stroke dementia was recorded if dementia was a listed diagnosis in the primary care record at the time of the index stroke. Pre-stroke dementia was excluded when there was no listed dementia diagnosis and baseline cognitive testing was above the cut-off for dementia (see below). For the remaining cases, pre-stroke dementia diagnosis was made by Dr. Sarah T. Pendlebury (a senior geriatrician with expertise in dementia) after reviewing all available study assessment data and hand-searching of the entire primary care record, including individual consultation records, all hospital outpatient clinic letters, and hospitalization documentation to establish pre-stroke dementia diagnosis on the basis of the *Diagnostic and Statistical Manual of Mental Disorders*, 4^th^ Edition (DSM-IV) criteria and by IQCODE score ≥3.6. Post-stroke dementia diagnosis was made after exclusion of patients with pre-stroke dementia, and required MMSE<24[^11^](#_ENREF_11) (remaining <24 for all subsequent follow-ups) or MoCA<20 or TICSm<22 or T-MoCA<9.[^6^](#_ENREF_6) For subjects with an incomplete test (i.e. testing performed but there was a problem like dysphasia, visual impairment, inability to use the dominant arm, or English as a second language that interfered with test completion), individual patient scores were reviewed, and patients with cognitive scores above cut-off were designated as no-dementia. For those scoring below cut-off, individual patient study records including those from primary care and informant-derived information were used to determine whether the DSM-IV criteria were met, to avoid spurious classifications on the basis of a low cognitive score. For patients without a direct study assessment, post-stroke dementia was diagnosed if a diagnosis of dementia was recorded in the primary care record, or if DSM-IV criteria[^12^](#_ENREF_12) were met after hand-searching of the entire primary care record as described by Kokmen et al[^13^](#_ENREF_13) and dementia was listed on the death certificate. Previous assessments have established that there is no under-diagnosis of dementia in OXVASC using this methodology.[^14^](#_ENREF_14)

**References**

1. Folstein MF, Folstein SE, McHugh PR. "Mini-mental state". A practical method for grading the cognitive state of patients for the clinician. *J Psychiatr Res* 1975; **12**(3): 189-98.

2. Nasreddine ZS, Phillips NA, Bedirian V, et al. The Montreal Cognitive Assessment, MoCA: a brief screening tool for mild cognitive impairment. *Journal of the American Geriatrics Society* 2005; **53**(4): 695-9.

3. Brandt J, Spencer M, Folstein MF. The Telephone Interview for Cognitive Status. *Neuropsychiatry Neuropsychol Behavioral Neurol* 1988; **1**(2): 111-7.

4. Hachinski V, Iadecola C, Petersen RC, et al. National Institute of Neurological Disorders and Stroke-Canadian Stroke Network vascular cognitive impairment harmonization standards. *Stroke* 2006; **37**(9): 2220-41.

5. Pendlebury ST, Mariz J, Bull L, Mehta Z, Rothwell PM. MoCA, ACE-R, and MMSE versus the National Institute of Neurological Disorders and Stroke-Canadian Stroke Network Vascular Cognitive Impairment Harmonization Standards Neuropsychological Battery after TIA and stroke. *Stroke* 2012; **43**(2): 464-9.

6. Pendlebury ST, Welch SJ, Cuthbertson FC, Mariz J, Mehta Z, Rothwell PM. Telephone assessment of cognition after transient ischemic attack and stroke: modified telephone interview of cognitive status and telephone Montreal Cognitive Assessment versus face-to-face Montreal Cognitive Assessment and neuropsychological battery. *Stroke* 2013; **44**(1): 227-9.

7. Hodkinson HM. Evaluation of a mental test score for assessment of mental impairment in the elderly. *Age and ageing* 1972; **1**(4): 233-8.

8. Pendlebury ST, Klaus SP, Mather M, de Brito M, Wharton RM. Routine cognitive screening in older patients admitted to acute medicine: abbreviated mental test score (AMTS) and subjective memory complaint versus Montreal Cognitive Assessment and IQCODE. *Age and ageing* 2015; **44**(6): 1000-5.

9. Pendlebury ST, Cuthbertson FC, Welch SJ, Mehta Z, Rothwell PM. Underestimation of cognitive impairment by Mini-Mental State Examination versus the Montreal Cognitive Assessment in patients with transient ischemic attack and stroke: a population-based study. *Stroke* 2010; **41**(6): 1290-3.

10. Jorm AF. A short form of the Informant Questionnaire on Cognitive Decline in the Elderly (IQCODE): development and cross-validation. *Psychological medicine* 1994; **24**(1): 145-53.

11. Mitchell AJ. A meta-analysis of the accuracy of the mini-mental state examination in the detection of dementia and mild cognitive impairment. *J Psychiatr Res* 2009; **43**(4): 411-31.

12. American Psychiatric Association. Diagnostic and Statistical Manual of Mental Disorders (DSM-IV). 4 ed. Washington, DC: American Psychiatric Association, 24 International Standard Classification; 1994.

13. Kokmen E, Whisnant JP, O'Fallon WM, Chu CP, Beard CM. Dementia after ischemic stroke: a population-based study in Rochester, Minnesota (1960-1984). *Neurology* 1996; **46**(1): 154-9.

14. Pendlebury ST, Chen PJ, Bull L, et al. Methodological factors in determining rates of dementia in transient ischemic attack and stroke: (I) impact of baseline selection bias. *Stroke* 2015; **46**(3): 641-6.

15. van Swieten JC, Koudstaal PJ, Visser MC, Schouten HJ, van Gijn J. Interobserver agreement for the assessment of handicap in stroke patients. *Stroke* 1988; **19**(5): 604-7.

**Web Appendix 4. Theoretical examples of non-linear weights for the modified Rankin Scale (mRS), derived from linear spline with knots at (A) 1 and 2, (B) 2 and 3, or (C) 3 and 4, with corresponding coefficients of 1, 4, and 2 for the resulting three segments (y over x1, x2, x3).** As seen from the shapes of the figures, this results in unequal “distances” between health-states in the earlier versus latter portions of the mRS, with the distance being greatest (step-change) between the two knots.

**
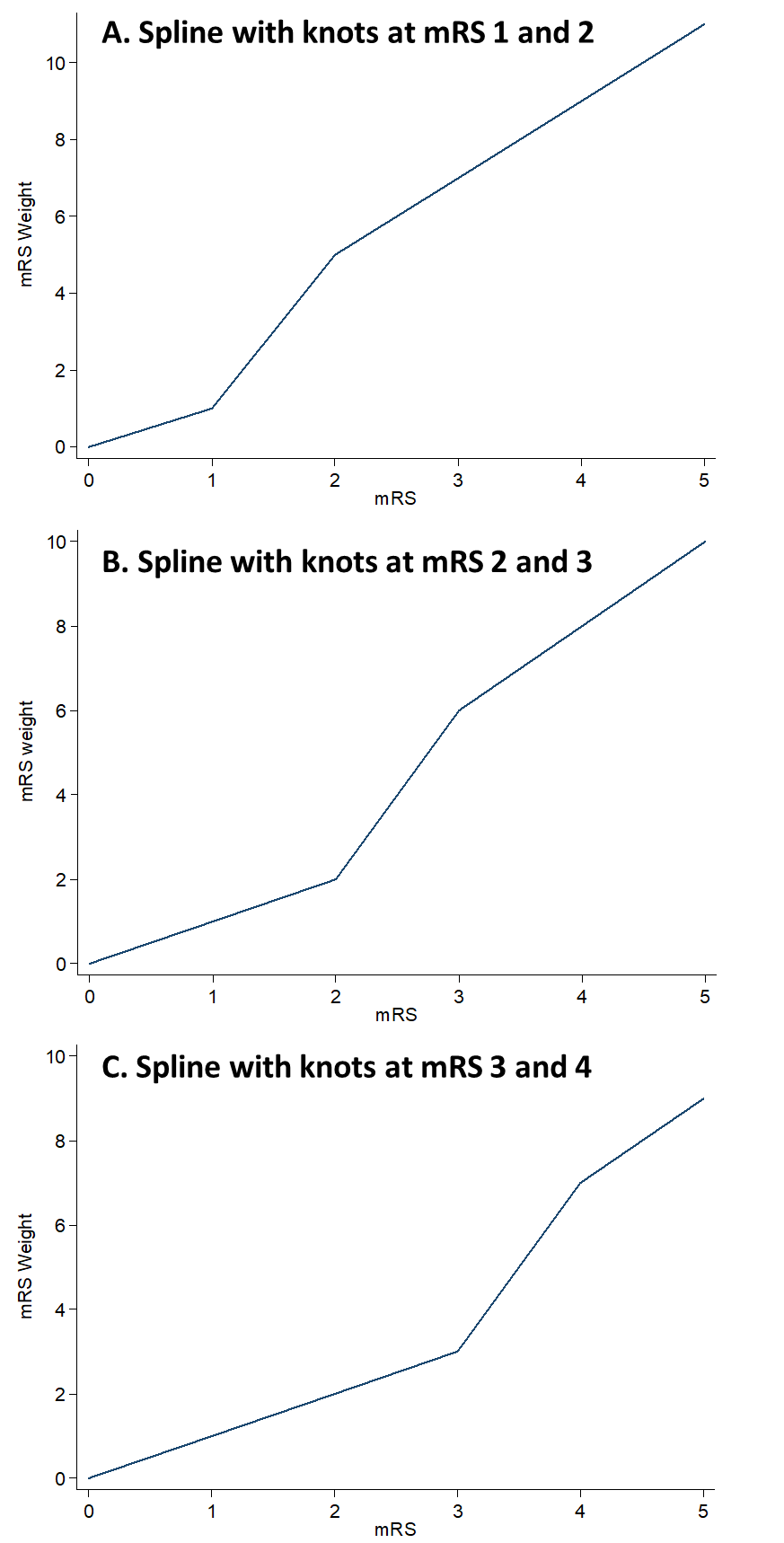
**

**Web Appendix 5. Patient sample and characteristics for 3-month survivors of ischaemic stroke (n=1,426)**

|  | **N** |
| --- | --- |
| **Age, mean (S.D.)** | 73.2 (12.7) |
| **Sex – male (%)** | 753 (52.8) |
| **Previous history (%):** |  |
| MI | 177 (12.4) |
| Angina | 239 (16.8) |
| Atrial Fibrillation | 260 (18.2) |
| Hypertension | 889 (62.4) |
| Dyslipidemia | 469 (32.9) |
| Diabetes | 205 (14.4) |
| PVD | 108 (7.6) |
| Stroke | 158 (11.1) |
| TIA | 205 (14.4) |
| Smoking | 836 (58.6) |
| Cancer | 22 (15.4) |
| Prior disability: mRS >2 | 243 (17.0) |
| Prior disability: mRS >1 | 433 (30.4) |
| Pre-stroke dementia | 144 (10.1) |
| Pre-stroke Institutionalization | 42 (2.9) |

**Web Appendix 6. Flow diagram for patients with ischaemic stroke occurring from April 2002 to March 2014 in the Oxford Vascular Study, followed until 15-May-2017**

**
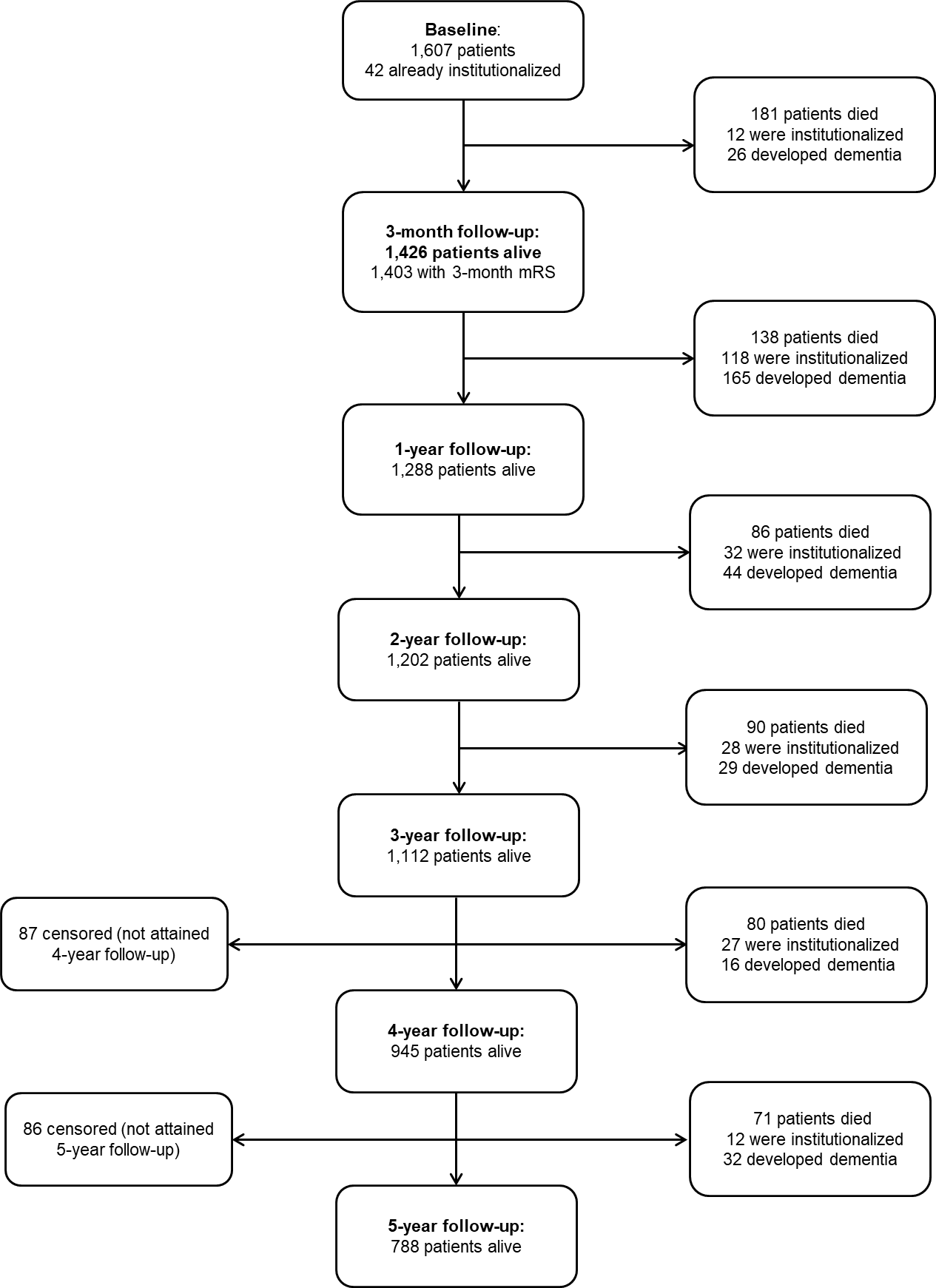
**

**Web Appendix 7. Representative plots of 5-year outcomes – death (A-B) and health/social care costs (C-D) –** **against the 3-month mRS using its standard values (A,D) versus using the theoretical weights shown in Web Appendix 5** Note that using weighted mRS results in a much better-fitting linear trendline in each case.

**
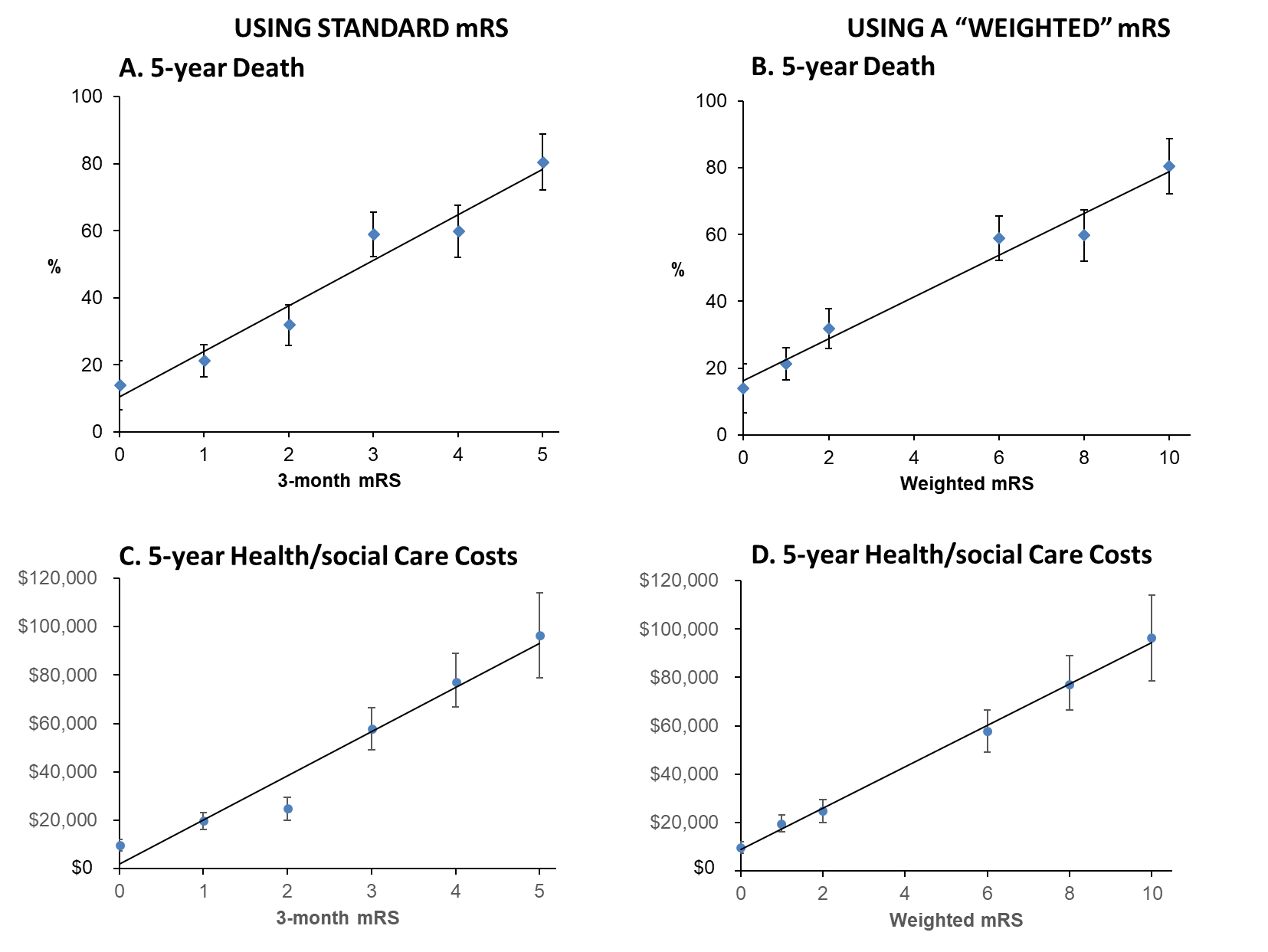
**

**Web Appendix 8.**

**Root mean squared error (RMSE) for linear regression of various 5-year outcomes (dependent variable) on the 3-month mRS (independent variable) in 3-month survivors of ischaemic stroke, with the mRS plotted as a linear scale (0, 1, 2, 3, 4, 5) versus also incorporating a spline (and thus a weighted step-change) at transitions 1-2, or 2-3, or 3-4. The difference in RMSE in each case compared to using the linear mRS (∆RMSE) is also shown, expressed as a percentage of the RMSE obtained using the linear mRS.**

| **Outcome** | **Unweighted mRS** | **Weighted with**  **step-change at 1-2** | | **Weighted with**  **step-change at 2-3** | | **Weighted with**  **step-change at 3-4** | |
| --- | --- | --- | --- | --- | --- | --- | --- |
|  | **RMSE (Reference)** | **RMSE** | **∆RMSE (%)** | **RMSE** | **∆RMSE (%)** | **RMSE** | **∆RMSE (%)** |
| **Death (%)** | 4.90 | 5.71 | **+16.5** | 2.92 | **-40.4** | 8.71 | **+77.8** |
| **Death/institutionalization (%)** | 5.82 | 7.33 | **+25.9** | 2.99 | **-48.6** | 10.3 | **+77.0** |
| **Death/dementia (%)** | 5.58 | 5.56 | **-0.4** | 4.58 | **-17.9** | 10.8 | **+93.5** |
| **Death/institutionalization/**  **Dementia (%)** | 5.53 | 6.22 | **+12.5** | 4.06 | **-26.6** | 11.0 | **+98.9** |
| **5-year Costs ($)** | 6,729 | 9,734 | **+44.7** | 1,744 | **-74.1** | 8,079 | **+20.1** |
| **5-year QALE (years)** | 0.21 | 0.32 | **+52.4** | 0.14 | **-33.3** | 0.34 | **+61.9** |

**Web Appendix 9. Odds and probability-weights for 5-year death, dementia, or institutionalization for each 3-month mRS score (age/sex-adjusted) in 3-month ischaemic stroke survivors with full 5-years of follow-up, or who met the endpoint of interest between 3-months and 5-years.** For the regressions of dementia and institutionalization, we excluded those who had a pre-stroke diagnosis of dementia and were institutionalized pre-stroke, respectively. All probabilities (Pr) were estimated from the logistic regressions and are presented to two significant figures.

|  | **Death at 5-years** | | **Dementia at 5-years**  **(or prior to death)** | | **Death or Dementia** | | **Institutionalization at 5-years (or prior to death)** | | **Death or**  **Institutionalization** | | **Death, Dementia, or Institutionalization** | |
| --- | --- | --- | --- | --- | --- | --- | --- | --- | --- | --- | --- | --- |
| **3-month mRS** | **aOR (95%CI)** | **Pr**  **(95%CI)** | **aOR**  **(95%CI)** | **Pr**  **(95%CI)** | **aOR (95%CI)** | **Pr**  **(95%CI)** | **aOR**  **(95%CI)** | **Pr**  **(95%CI)** | **aOR (95%CI)** | **Pr**  **(95%CI)** | **aOR (95%CI)** | **Pr**  **(95%CI)** |
|  |  |  |  |  |  |  |  |  |  |  |  |  |
| **0** | **Reference** | **0.13**  (0.11-0.15) | **Reference** | **0.11**  (0.099-0.12) | **Reference** | **0.19**  (0.17-0.21) | **Reference** | **0.019**  (0.016-0.022) | **Reference** | **0.13**  (0.11-0.15) | **Reference** | **0.19**  (0.16-0.22) |
| **1** | 1.20  (0.62-2.31) | **0.19**  (0.18-0.20) | 0.86  (0.43-1.73) | **0.11**  (0.10-0.12) | 1.16  (0.66-2.04) | **0.25**  (0.23-0.27) | 2.96  (0.68-12.9) | **0.065**  (0.060-0.070) | 1.57  (0.81-3.01) | **0.22**  (0.20-0.24) | 1.31  (0.74-2.32) | **0.27**  (0.25-0.29) |
| **2** | 1.64  (0.85-3.17) | **0.28**  (0.26-0.30) | 1.61  (0.81-3.19) | **0.21**  (0.20-0.22) | 1.92  (1.09-3.40) | **0.40**  (0.38-0.42) | 2.81  (0.64-12.4) | **0.077**  (0.071-0.083) | 1.94  (1.00-3.75) | **0.32**  (0.30-0.34) | 1.99  (1.12-3.55) | **0.41**  (0.38-0.44) |
| **3** | 3.81  (1.97-7.37) | **0.55**  (0.52-0.58) | 2.97  (1.50-5.88) | **0.38**  (0.36-0.40) | 4.96  (2.76-8.91) | **0.69**  (0.67-0.71) | 10.1  (2.39-43.1) | **0.28**  (0.26-0.30) | 6.21  (3.19-12.1) | **0.65**  (0.62-0.68) | 5.94  (3.26-10.8) | **0.73**  (0.71-0.75) |
| **4** | 4.59  (2.31-9.10) | **0.57**  (0.54-0.60) | 4.30  (2.11-8.77) | **0.47**  (0.44-0.50) | 5.87  (3.14-11.0) | **0.71**  (0.68-0.74) | 19.0  (4.44-81.0) | **0.41**  (0.38-0.44) | 8.57  (4.25-17.3) | **0.70** (0.67-0.73) | 8.31  (4.39-16.1) | **0.77**  (0.74-0.80) |
| **5** | 10.4  (4.83-22.6) | **0.74**  (0.71-0.77) | 11.5  (5.13-25.7) | **0.68**  (0.65-0.71) | 16.5  (7.41-36.6) | **0.87**  (0.85-0.89) | 44.2  (10.1-194) | **0.60**  (0.57-0.63) | 38.0  (15.1-95.3) | **0.90**  (0.88-0.92) | 43.2  (15.8-118) | **0.94**  (0.93-0.95) |
| **N** | 1235 |  | 1154 |  | 1255 |  | 1210 |  | 1244 |  | 1262 |  |
| **AUC** | **0.821** |  | **0.788** |  | **0.834** |  | **0.843** |  | **0.858** |  | **0.860** |  |
| **p>\|X^2^\|** |  | <0.0001* |  | <0.0001* |  | <0.0001* |  | <0.0001* |  | <0.0001* |  | <0.0001* |

**Web Appendix 10.** Age- and sex-adjusted probabilities estimated from logistic regressions for 1-year (dark blue), 2-year (red), 3-year (green), 4-year (purple), and 5-year (light blue) outcomes of (A) post-stroke institutionalization and (B) post-stroke dementia for 3-month survivors of ischaemic stroke (n=1,425), stratified by 3-month mRS. Bars represent 95% confidence intervals.

**
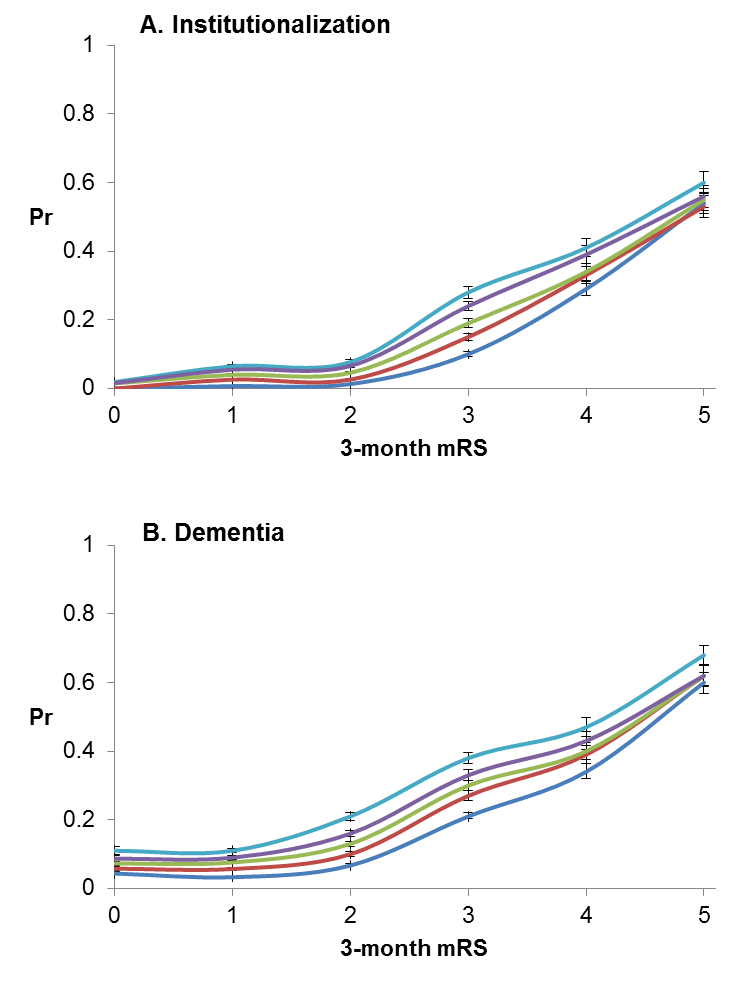
**We

**Web Appendix 11. Age- and sex-adjusted probabilities of death in Years 1 through 5, estimated from logistic regressions in 3-month survivors of ischaemic stroke, grouped by 3-month mRS.** All probabilities (Pr) are presented with two significant figures and alongside the number of individuals contributing to that estimate.

|  | **Year 1** | | **Year 2** | | **Year 3** | | **Year 4** | | **Year 5** | |
| --- | --- | --- | --- | --- | --- | --- | --- | --- | --- | --- |
| **3-month mRS** | **Pr**  **(95%CI)** | **N** | **Pr**  **(95%CI)** | **N** | **Pr**  **(95%CI)** | **N** | **Pr**  **(95%CI)** | **N** | **Pr**  **(95%CI)** | **N** |
| **0** | 0.015  (0.013-0.017) | 137 | 0.036  (0.032-0.040) | 137 | 0.058  (0.050-0.066) | 137 | 0.089  (0.076-0.10) | 124 | 0.13  (0.11-0.15) | 107 |
| **1** | 0.026  (0.024-0.028) | 427 | 0.047  (0.043-0.051) | 427 | 0.073  (0.067-0.079) | 427 | 0.13  (0.12-0.14) | 385 | 0.19  (0.18-0.20) | 356 |
| **2** | 0.030  (0.028-0.032) | 305 | 0.072  (0.067-0.077) | 305 | 0.14  (0.13-0.15) | 305 | 0.20  (0.18-0.22) | 290 | 0.28  (0.26-0.30) | 273 |
| **3** | 0.11  (0.10-0.12) | 251 | 0.24  (0.23-0.25) | 251 | 0.33  (0.31-0.35) | 251 | 0.44  (0.42-0.46) | 243 | 0.55  (0.52-0.58) | 234 |
| **4** | 0.22  (0.20-0.24) | 180 | 0.31  (0.29-0.33) | 180 | 0.42  (0.39-0.45) | 180 | 0.50  (0.47-0.53) | 174 | 0.57  (0.54-0.60) | 169 |
| **5** | 0.43  (0.40-0.46) | 103 | 0.51  (0.48-0.54) | 103 | 0.60  (0.57-0.63) | 103 | 0.67  (0.64-0.70) | 103 | 0.74  (0.71-0.77) | 96 |
| **Total N** |  | 1403 |  | 1403 |  | 1403 |  | 1319 |  | 1235 |
| **AUC (95%CI)** | 0.81 (0.77-0.84) |  | 0.83 (0.80-0.86) |  | 0.83 (0.81-0.86) |  | 0.84 (0.82-0.86) |  | 0.84 (0.82-0.86) |  |
| **Hosmer-Lemeshow chi^2^** | 4.24 P=0.83* ***Non-significant P is desired and indicates good model fit** | | 11.25 | P=0.19 | 5.20 | P=0.74 | 8.61 | P=0.38 | 11.74 | P=0.16 |

**Web Appendix 12. Age- and sex-adjusted probabilities of death or post-stroke dementia in Years 1 through 5, estimated from logistic regressions in 3-month survivors of ischaemic stroke, grouped by 3-month mRS.** All probabilities (Pr) are presented with two significant figures and alongside the number of individuals contributing to that estimate.

|  | **Year 1** | | **Year 2** | | **Year 3** | | **Year 4** | | **Year 5** | |
| --- | --- | --- | --- | --- | --- | --- | --- | --- | --- | --- |
| **3-month mRS** | **Pr**  **(95%CI)** | **N** | **Pr**  **(95%CI)** | **N** | **Pr**  **(95%CI)** | **N** | **Pr**  **(95%CI)** | **N** | **Pr**  **(95%CI)** | **N** |
| **0** | 0.058  (0.052-0.064) | 137 | 0.080  (0.072-0.088) | 137 | 0.11  (0.098-0.12) | 137 | 0.14  (0.12-0.16) | 127 | 0.19  (0.17-0.21) | 112 |
| **1** | 0.054  (0.051-0.057) | 427 | 0.096  (0.090-0.10) | 427 | 0.13  (0.12-0.14) | 427 | 0.19  (0.18-0.20) | 385 | 0.25  (0.23-0.27) | 359 |
| **2** | 0.092  (0.087-0.097) | 305 | 0.15  (0.14-0.16) | 305 | 0.23  (0.22-0.24) | 305 | 0.29  (0.27-0.31) | 292 | 0.40  (0.38-0.42) | 275 |
| **3** | 0.27  (0.26-0.28) | 251 | 0.42  (0.40-0.44) | 251 | 0.49  (0.47-0.51) | 251 | 0.58  (0.56-0.60) | 248 | 0.69  (0.67-0.71) | 242 |
| **4** | 0.43  (0.41-0.45) | 180 | 0.52  (0.50-0.54) | 180 | 0.59  (0.56-0.62) | 180 | 0.64  (0.61-0.67) | 174 | 0.71  (0.68-0.74) | 170 |
| **5** | 0.66  (0.64-0.68) | 103 | 0.71  (0.68-0.74) | 103 | 0.75  (0.72-0.78) | 103 | 0.78  (0.75-0.81) | 103 | 0.87  (0.85-0.89) | 97 |
| **Total N** |  | 1403 |  | 1403 |  | 1403 |  | 1329 |  | 1255 |
| **AUC (95%CI)** | 0.80 (0.78-0.83) |  | 0.82 (0.79-0.84) |  | 0.83 (0.81-0.85) |  | 0.83 (0.81-0.85) |  | 0.85 (0.83-0.87) |  |
| **Hosmer-Lemeshow chi^2^** | 1.95 | P=0.98 | 8.31 | P-=0.40 | 4.95 | P=0.76 | 6.37 | P=0.61 | 3.78 | P=0.88 |

**Web Appendix 13. Age- and sex-adjusted probabilities of death or post-stroke institutionalization in Years 1 through 5, estimated from logistic regressions in 3-month survivors of ischaemic stroke, grouped by 3-month mRS.** All probabilities (Pr) are presented with two significant figures and alongside the number of individuals contributing to that estimate.

|  | **Year 1** | | | **Year 2** | | **Year 3** | | **Year 4** | | **Year 5** | |
| --- | --- | --- | --- | --- | --- | --- | --- | --- | --- | --- | --- |
| **3-month mRS** | **Pr**  **(95%CI)** | | **N** | **Pr**  **(95%CI)** | **N** | **Pr**  **(95%CI)** | **N** | **Pr**  **(95%CI)** | **N** | **Pr**  **(95%CI)** | **N** |
| **0** | 0.015  (0.013-0.017) | | 137 | 0.036  (0.031-0.041) | 137 | 0.066  (0.056-0.076) | 137 | 0.089  (0.075-0.10) | 124 | 0.13  (0.11-0.15) | 107 |
| **1** | 0.033  (0.030-0.036) | | 427 | 0.070  (0.064-0.076) | 427 | 0.10  (0.091-0.11) | 427 | 0.17  (0.16-0.18) | 385 | 0.22  (0.20-0.24) | 356 |
| **2** | 0.039  (0.036-0.042) | | 305 | 0.085  (0.078-0.092) | 305 | 0.16  (0.15-0.17) | 305 | 0.24  (0.22-0.26) | 290 | 0.32  (0.30-0.34) | 273 |
| **3** | 0.18  (0.17-0.19) | | 251 | 0.33  (0.31-0.35) | 251 | 0.44  (0.42-0.46) | 251 | 0.56  (0.53-0.59) | 243 | 0.65  (0.62-0.68) | 234 |
| **4** | 0.42  (0.39-0.45) | | 180 | 0.53  (0.50-0.56) | 180 | 0.57  (0.54-0.60) | 180 | 0.64  (0.61-0.67) | 174 | 0.70 (0.67-0.73) | 169 |
| **5** | 0.75  (0.72-0.78) | | 103 | 0.77  (0.74-0.80) | 103 | 0.82  (0.79-0.85) | 103 | 0.85  (0.82-0.88) | 103 | 0.90  (0.88-0.92) | 96 |
| **Total N** |  | | 1403 |  | 1403 |  | 1403 |  | 1319 |  | 1235 |
| **AUC (95%CI)** | 0.84 (0.81-0.86) |  | | 0.85 (0.82-0.87) |  | 0.85 (0.83-0.87) |  | 0.86 (0.84-0.88) |  | 0.86 (0.84-0.88) |  |
| **Hosmer-Lemeshow chi^2^** | 5.82 | P=0.67 | | 11.62 | P=0.17 | 4.43 | P=0.82 | 9.43 | P=0.31 | 14.47 | P=0.070 |

**Web Appendix 14. Age- and sex-adjusted probabilities of death, post-stroke dementia, or institutionalization in Years 1 through 5, estimated from logistic regressions in 3-month survivors of ischaemic stroke, grouped by 3-month mRS.** All probabilities (Pr) are presented with two significant figures and alongside the number of individuals contributing to that estimate.

|  | **Year 1** | | **Year 2** | | **Year 3** | | **Year 4** | | **Year 5** | |
| --- | --- | --- | --- | --- | --- | --- | --- | --- | --- | --- |
| **3-month mRS** | **Pr**  **(95%CI)** | **N** | **Pr**  **(95%CI)** | **N** | **Pr**  **(95%CI)** | **N** | **Pr**  **(95%CI)** | **N** | **Pr**  **(95%CI)** | **N** |
| **0** | 0.058  (0.051-0.065) | 137 | 0.080  (0.070-0.090) | 137 | 0.11  (0.096-0.12) | 137 | 0.14  (0.12-0.16) | 127 | 0.19  (0.16-0.22) | 112 |
| **1** | 0.061  (0.057-0.065) | 427 | 0.11  (0.10-0.12) | 427 | 0.16  (0.15-0.17) | 427 | 0.22  (0.20-0.24) | 386 | 0.27  (0.25-0.29) | 360 |
| **2** | 0.098  (0.091-0.11) | 305 | 0.16  (0.15-0.17) | 305 | 0.25  (0.23-0.27) | 305 | 0.32  (0.30-0.34) | 292 | 0.41  (0.38-0.44) | 275 |
| **3** | 0.33  (0.31-0.35) | 251 | 0.49  (0.47-0.51) | 251 | 0.57  (0.55-0.59) | 251 | 0.64  (0.62-0.66) | 248 | 0.73  (0.71-0.75) | 244 |
| **4** | 0.53  (0.50-0.56) | 180 | 0.63  (0.60-0.66) | 180 | 0.66  (0.63-0.69) | 180 | 0.72  (0.69-0.75) | 176 | 0.77  (0.74-0.80) | 172 |
| **5** | 0.81  (0.79-0.83) | 103 | 0.83  (0.80-0.86) | 103 | 0.86  (0.84-0.88) | 103 | 0.89  (0.87-0.91) | 103 | 0.94  (0.93-0.95) | 99 |
| **Total N** |  | 1403 |  | 1403 |  | 1403 |  | 1332 |  | 1262 |
| **AUC (95%CI)** | 0.85 (0.83-0.87) |  | 0.85 (0.83-0.87) |  | 0.84 (0.82-0.86) |  | 0.86 (0.84-0.88) |  | 0.86 (0.84-0.88) |  |
| **Hosmer-Lemeshow chi^2^** | 5.99 | P=0.65 | 1.90 | P=0.98 | 12.81 | P=0.12 | 14.79 | P=0.063 | 10.36 | P=0.24 |

**Web Appendix 15.** Age- and sex-adjusted probabilities estimated from logistic regressions for 1-year (dark blue), 2-year (red), 3-year (green), 4-year (purple), and 5-year (light blue) outcomes of (A) death, (B) death or post-stroke dementia, (C) death or post-stroke institutionalization, and (D) death, dementia, or institutionalization for 3-month survivors of ischaemic stroke, stratified by 3-month mRS, excluding patients with pre-morbid mRS>2 (n=1,171).

**
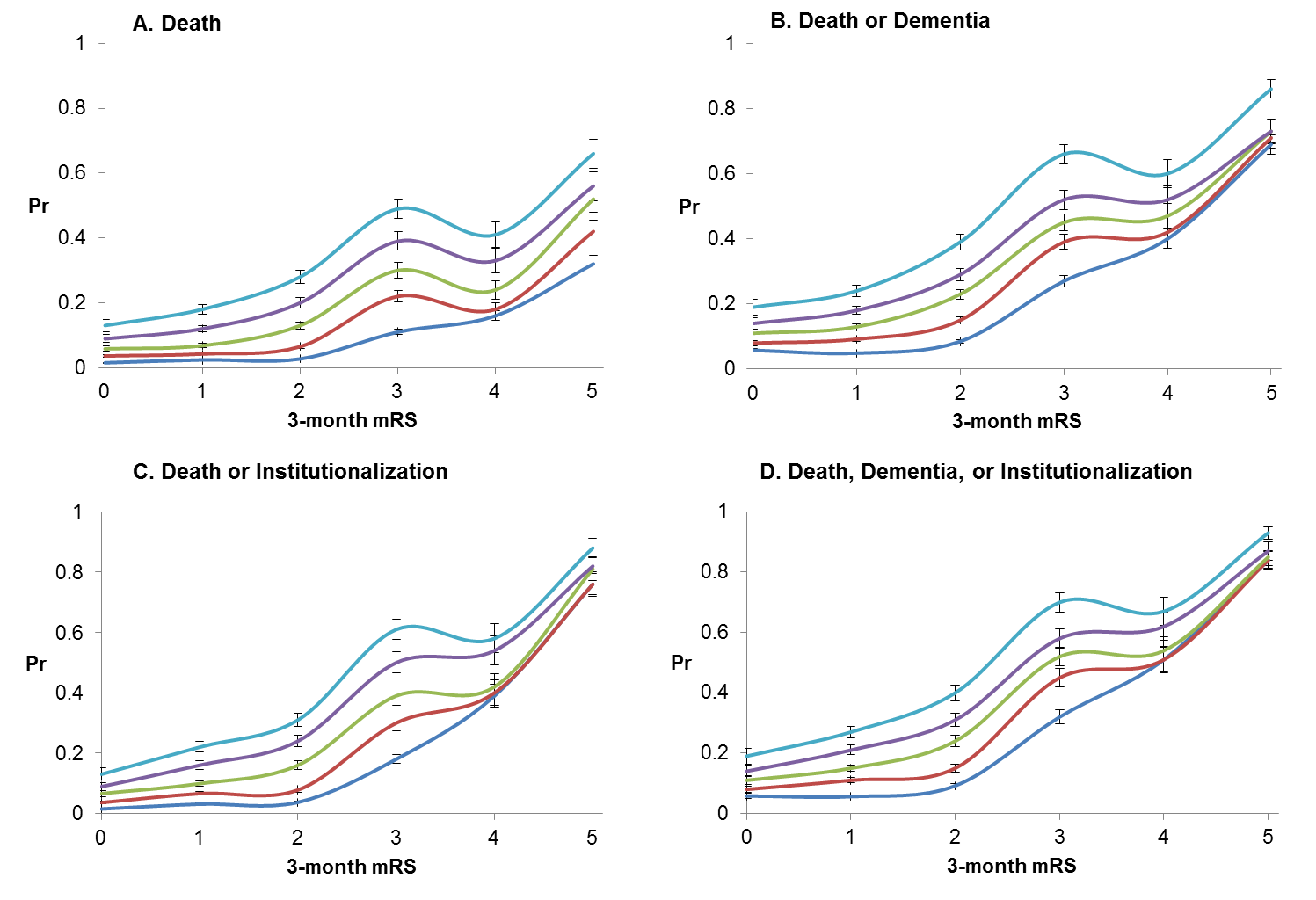
**

**Web Appendix 16.** Age- and sex-adjusted probabilities estimated from logistic regressions for 1-year (dark blue), 2-year (red), 3-year (green), 4-year (purple), and 5-year (light blue) outcomes of (A) death, (B) death or post-stroke dementia, (C) death or post-stroke institutionalization, and (D) death, dementia, or institutionalization for 3-month survivors of ischaemic stroke, stratified by 3-month mRS, excluding patients with pre-morbid mRS>1 (n=984).


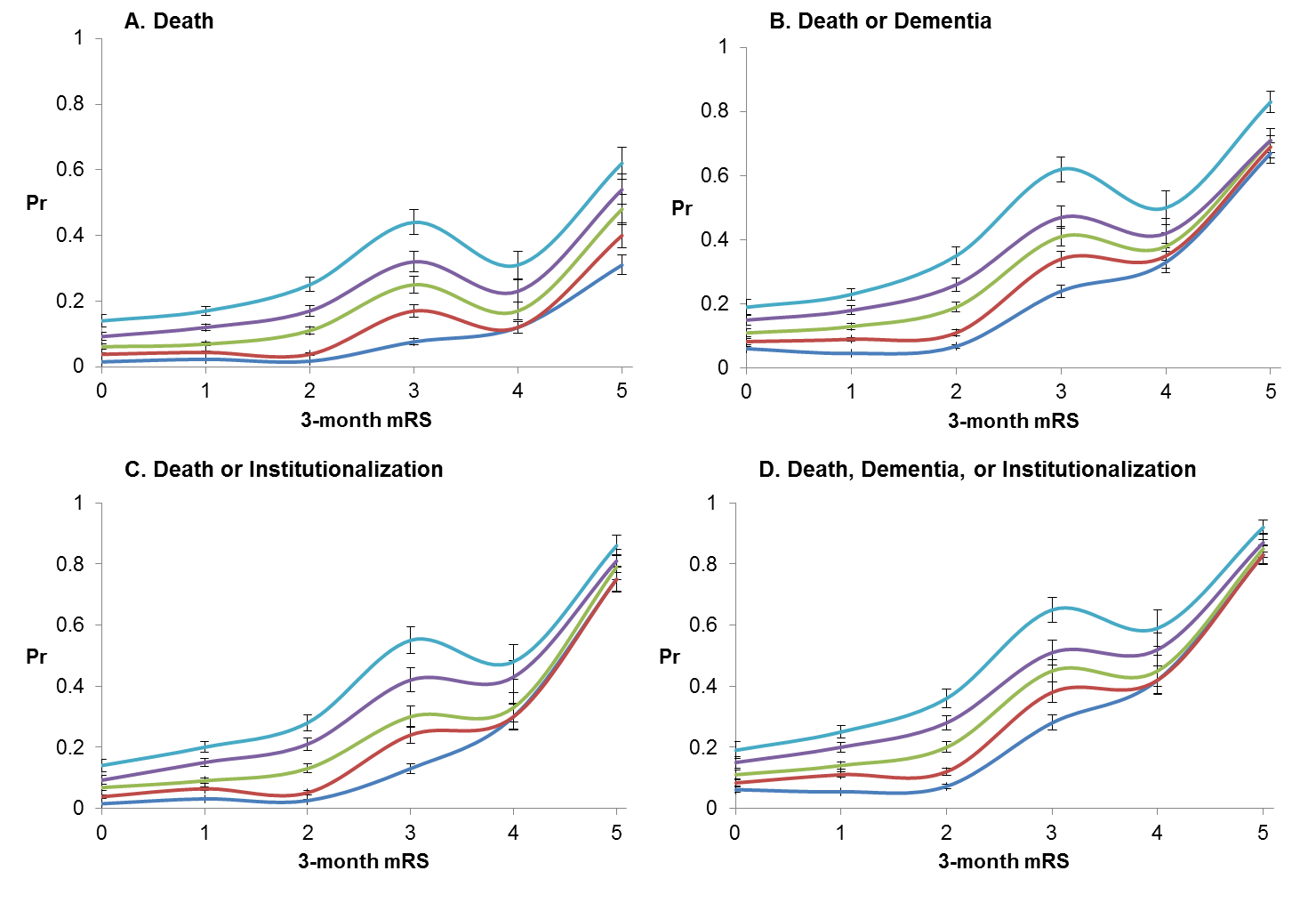


**Web Appendix 17.** Unadjusted probabilities estimated from logistic regressions for 1-year (dark blue), 2-year (red), 3-year (green), 4-year (purple), and 5-year (light blue) outcomes of (A) death, (B) death or post-stroke dementia, (C) death or post-stroke institutionalization, and (D) death, dementia, or institutionalization for 3-month survivors of ischaemic stroke, stratified by 3-month mRS, restricted to those aged <75 years (n=669).

**
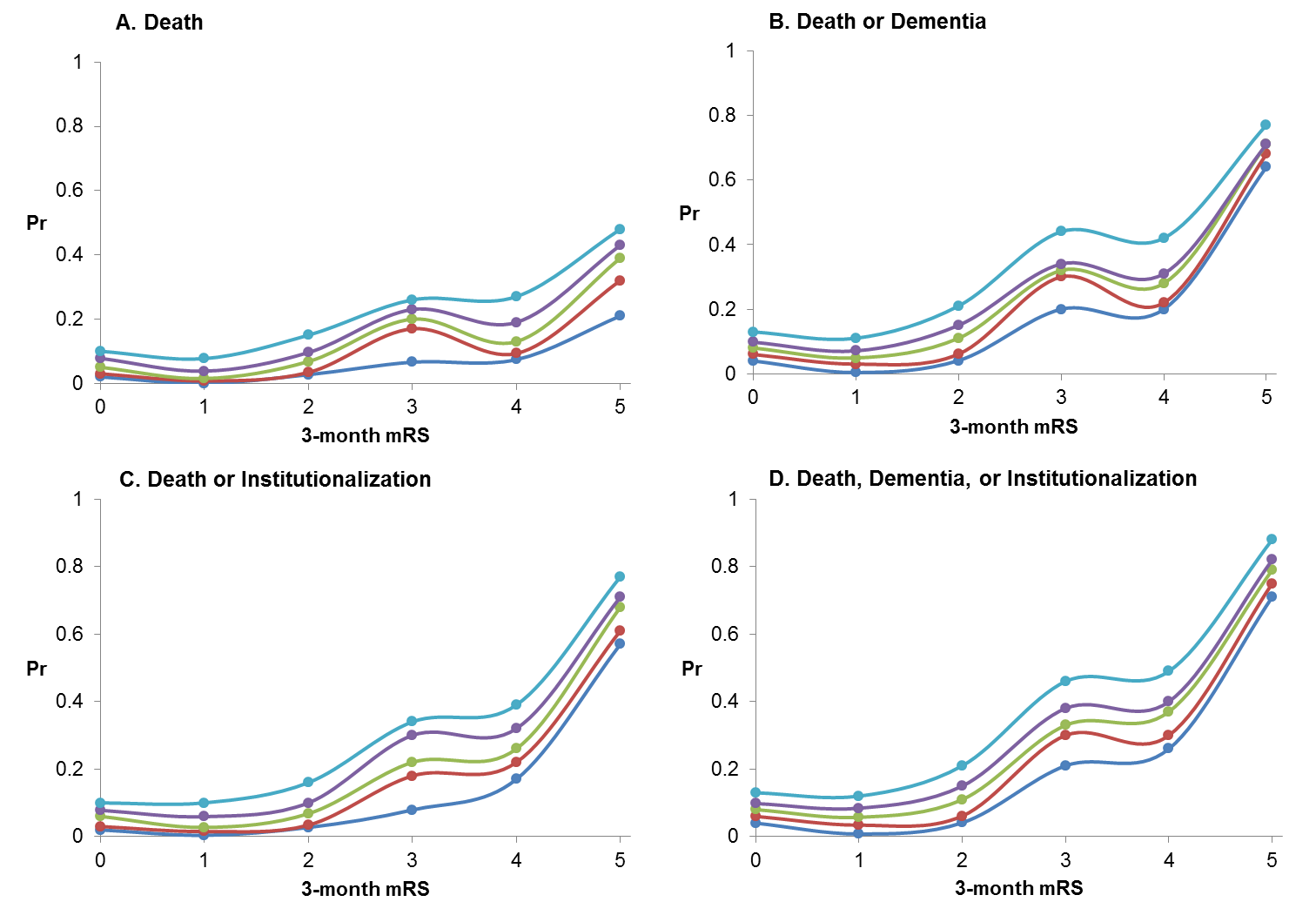
**

**Web Appendix 18.** Unadjusted probabilities estimated from logistic regressions for 1-year (dark blue), 2-year (red), 3-year (green), 4-year (purple), and 5-year (light blue) outcomes of (A) death, (B) death or post-stroke dementia, (C) death or post-stroke institutionalization, and (D) death, dementia, or institutionalization for 3-month survivors of ischaemic stroke, stratified by 3-month mRS, restricted to those aged >75 years (n=733).


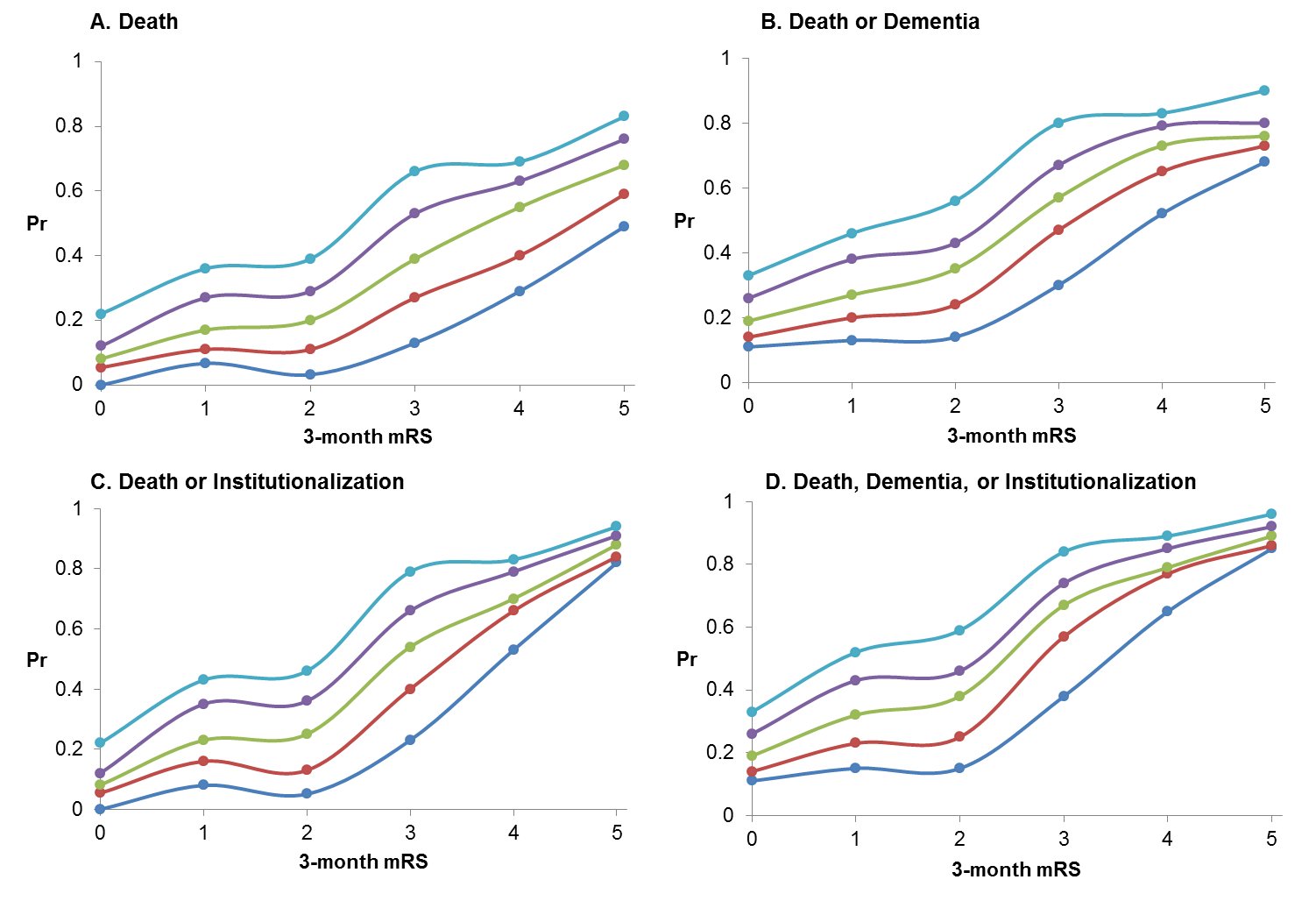


**Web Appendix 19.** Unadjusted probabilities estimated from logistic regressions for 1-year (dark blue), 2-year (red), 3-year (green), 4-year (purple), and 5-year (light blue) outcomes of (A) death, (B) death or post-stroke dementia, (C) death or post-stroke institutionalization, and (D) death, dementia, or institutionalization for 3-month survivors of ischaemic stroke, stratified by 3-month mRS, restricted to male sex (n=748).

**
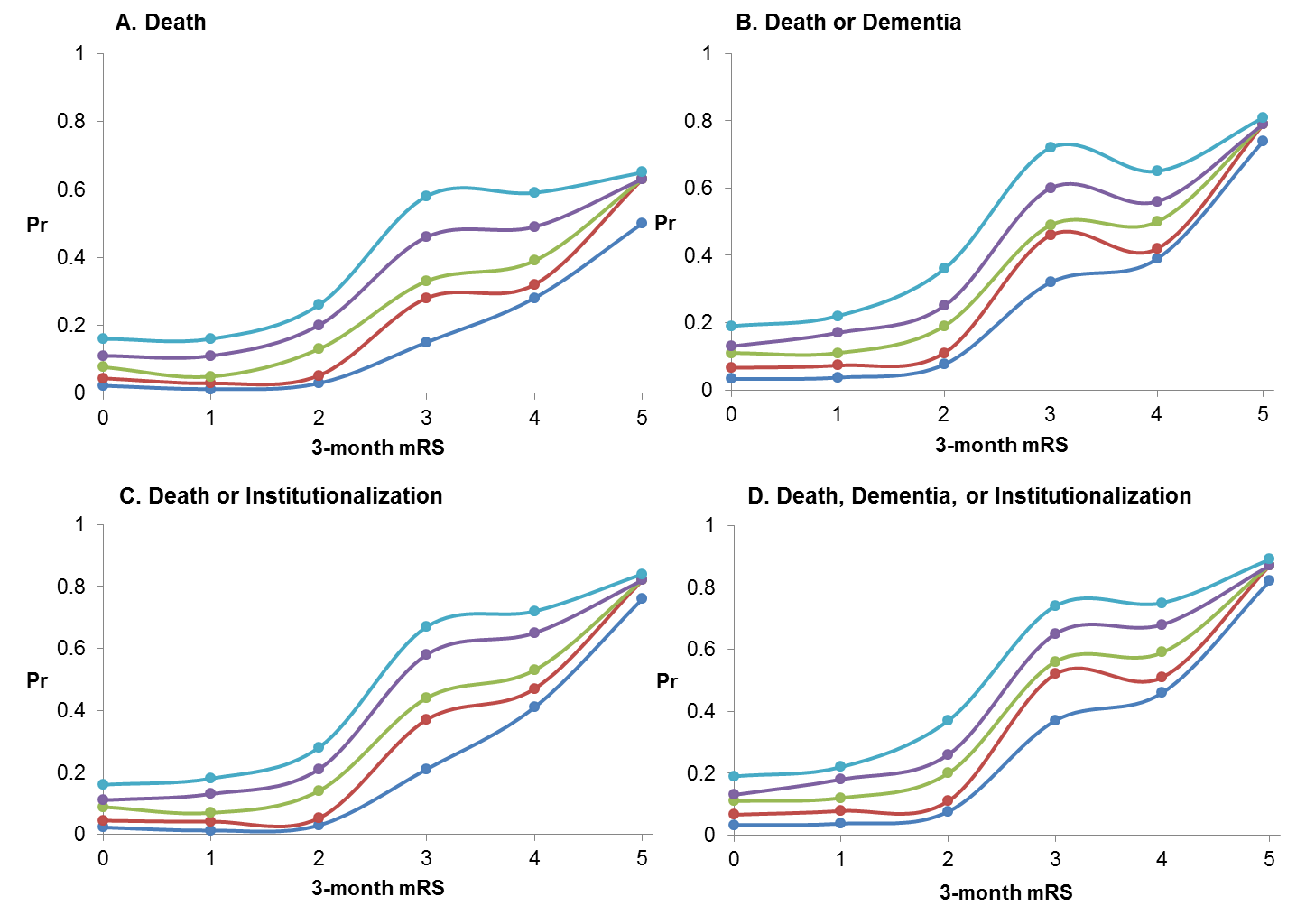
**

**Web Appendix 20.** Unadjusted probabilities estimated from logistic regressions for 1-year (dark blue), 2-year (red), 3-year (green), 4-year (purple), and 5-year (light blue) outcomes of (A) death, (B) death or post-stroke dementia, (C) death or post-stroke institutionalization, and (D) death, dementia, or institutionalization for 3-month survivors of ischaemic stroke, stratified by 3-month mRS, restricted to female sex (n=655).

**
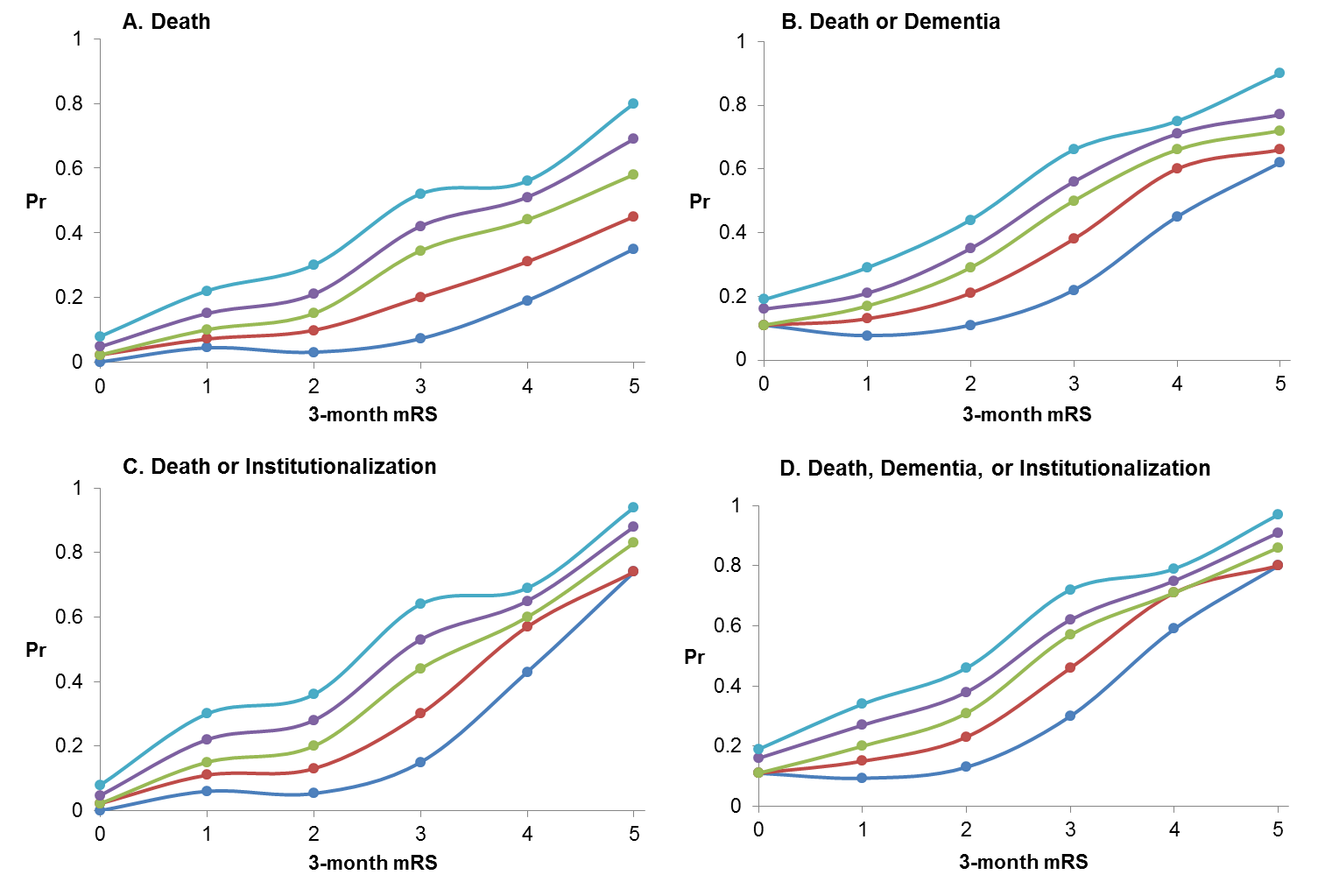
**

**Web Appendix 21. Comparison of probability weights for 5-year death, dementia, or post-stroke institutionalization, derived from logistic regressions in 3-month survivors of ischaemic stroke and grouped by 3-month mRS, in: all patients, excluding premorbid mRS>2, excluding premorbid mRS>1, age<75, age>75, men, women, excluding patients with recurrent strokes, and only those with recurrent strokes over follow-up.** All probabilities (Pr) are presented with two significant figures and alongside the number of individuals contributing to that estimate. Regressions in the first three groups were adjusted for age and sex. Below the probabilities for the 5 subgroups, the difference compared to the estimated probability for the overall cohort is presented (∆Pr = Pr for group – Pr for all patients). ∆Pr’s >0.10 or <-0.10, representing a >10% absolute difference in probability, are flagged with *.

|  | **All Patients** | | **Excluding Pre-morbid mRS>2** | | **Excluding Pre-morbid mRS>1** | | **Age <75 years** | | **Age >75 years** | | **Men** | | **Women** | | **Excluding Recurrent Events** | | **Recurrent Events Only** | |
| --- | --- | --- | --- | --- | --- | --- | --- | --- | --- | --- | --- | --- | --- | --- | --- | --- | --- | --- |
| **3-month mRS** | **Pr** | **N** | **Pr**  **(∆Pr)** | **N** | **Pr**  **(∆Pr)** | **N** | **Pr**  **(∆Pr)** | **N** | **Pr**  **(∆Pr)** | **N** | **Pr**  **(∆Pr)** | **N** | **Pr**  **(∆Pr)** | **N** | **Pr**  **(∆Pr)** | **N** | **Pr**  **(∆Pr)** | **N** |
|  |  |  |  |  |  |  |  |  |  |  |  |  |  |  |  |  |  |  |
| **0** | 0.19 | 112 | 0.19  (0.00) | 112 | 0.19  (0.00) | 108 | 0.13  (-0.06) | 82 | 0.33  (0.14*) | 30 | 0.19  (0.00) | 70 | 0.19  (0.00) | 42 | 0.16  (-0.03) | 88 | 0.29  (0.10) | 24 |
| **1** | 0.27 | 360 | 0.27  (0.00) | 358 | 0.25  (-0.02) | 326 | 0.12  (-0.15*) | 221 | 0.52  (0.25*) | 139 | 0.22  (-0.05) | 210 | 0.34  (0.07) | 150 | 0.25  (-0.02) | 282 | 0.36  (0.09) | 78 |
| **2** | 0.41 | 275 | 0.40  (-0.01) | 265 | 0.36  (-0.05) | 213 | 0.21  (-0.20*) | 128 | 0.59  (0.18*) | 147 | 0.37  (-0.04) | 152 | 0.46  (0.05) | 123 | 0.37  (-0.04) | 175 | 0.49  (0.08) | 100 |
| **3** | 0.73 | 244 | 0.70  (-0.03) | 152 | 0.65  (-0.08) | 101 | 0.46  (-0.27*) | 71 | 0.84  (0.11*) | 173 | 0.74  (0.01) | 122 | 0.72  (-0.01) | 122 | 0.70  (-0.03) | 172 | 0.81  (0.08) | 72 |
| **4** | 0.77 | 172 | 0.67  (-0.10) | 89 | 0.59  (-0.18*) | 64 | 0.49  (-0.28*) | 49 | 0.89  (0.12*) | 123 | 0.75  (-0.02) | 71 | 0.79  (0.02) | 101 | 0.73  (-0.04) | 127 | 0.89  (0.12*) | 45 |
| **5** | 0.94 | 99 | 0.93  (-0.01) | 59 | 0.92  (-0.02) | 50 | 0.88  (-0.06) | 26 | 0.96  (0.02) | 72 | 0.89  (-0.05) | 37 | 0.97  (0.03) | 62 | 0.95  (0.01) | 79 | 0.90  (-0.04) | 20 |
| **Total N** |  | 1262 |  | 1035 |  | 862 |  | 577 |  | 684 |  | 662 |  | 600 |  | 923 |  | 339 |

**Web Appendix 22. Estimated total health and social care costs at 1-year, 2-years, 3-years, 4-years, and 5-years post-stroke for each 3-month mRS score in 3-month ischaemic stroke survivors.**

| **3-month** | **Year 1** | **Year 2** | **Year 3** | **Year 4** | **Year 5** |
| --- | --- | --- | --- | --- | --- |
| **mRS** | **$ (95%CI)** | **$ (95%CI)** | **$ (95%CI)** | **$ (95%CI)** | **$ (95%CI)*** |
|  |  |  |  |  |  |
| **0** | 1,596  (1,125-2,113) | 3,812  (2,738-5,139) | 5,863  (4,239-7,988) | 7,716  (5,850-9,933) | 9,692  (7,486-12,037) |
| **1** | 2,266  (1,799-2,785) | 6,113  (5,039-7,285) | 10,265  (8,457-12,312) | 14,256  (11,804-17,077) | 19,539  (16,133-23,242) |
| **2** | 3,776  (2,928-4,664) | 8,594  (6,767-10,447) | 13,921  (11,208-16,851) | 19,653  (16,035-23,438) | 24,643  (20,115-29,436) |
| **3** | 11,197  (9,063-13,327) | 21,914  (18,227-25,680) | 34,170  (28,966-39,805) | 45,952  (39,225-53,434) | 57,595  (49,150-66,445) |
| **4** | 25,523  (22,315-29,050) | 40,800  (35,306-46,310) | 55,450  (47,695-63,518) | 66,369  (57,074-75,876) | 77,071  (66,663-88,926) |
| **5** | 36,946  (32,308-42,070) | 56,228  (48,109-64,298) | 72,368  (60,883-84,221) | 84,963  (70,027-99,936) | 96,343  (78,692-113,998) |
| **N** | 1403 | 1403 | 1403 | 1319 | 1235 |

Mean difference in costs at 5 years for mRS 0 vs 1: $9,847 (95%CI 5,814-14,174), mRS 1 vs 2: $5,104 (-1,090-10,614), mRS 2 vs 3: $32,952 (23,594-43,464), mRS 3 vs 4: $19,476 (5,700-33,318), mRS 4 vs 5: $19,272 (-1,141-40,510).

**Web Appendix 23.** Estimated (A) 5-year health and social care costs and (B) quality-adjusted life expectancy (in quality-adjusted life-years, QALYs) for 1-year (dark blue), 2-years (red), 3-years (green), 4-years (purple), and 5-years (light blue) post-stroke for 3-month survivors of ischaemic stroke, stratified by 3-month mRS, excluding those with pre-morbid mRS>2 (n=1,171).

**
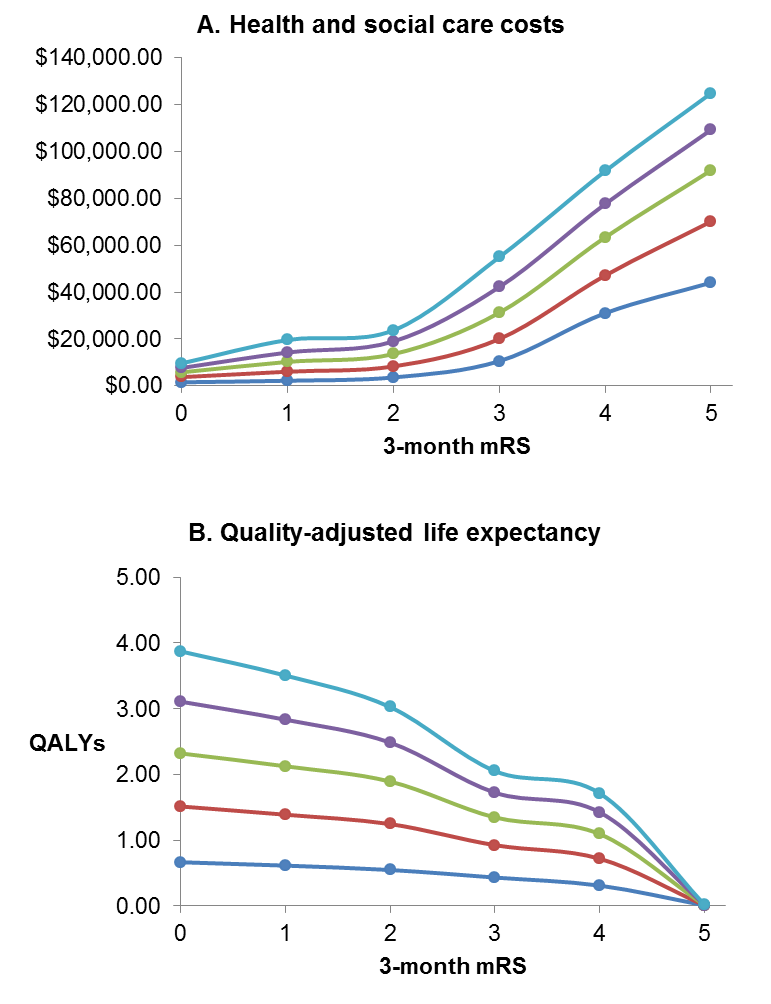
**

**Web Appendix 24.** Estimated (A) 5-year health and social care costs and (B) quality-adjusted life expectancy (in quality-adjusted life-years, QALYs) for 1-year (dark blue), 2-years (red), 3-years (green), 4-years (purple), and 5-years (light blue) post-stroke for 3-month survivors of ischaemic stroke, stratified by 3-month mRS, excluding those with pre-morbid mRS>1 (n=984).

**
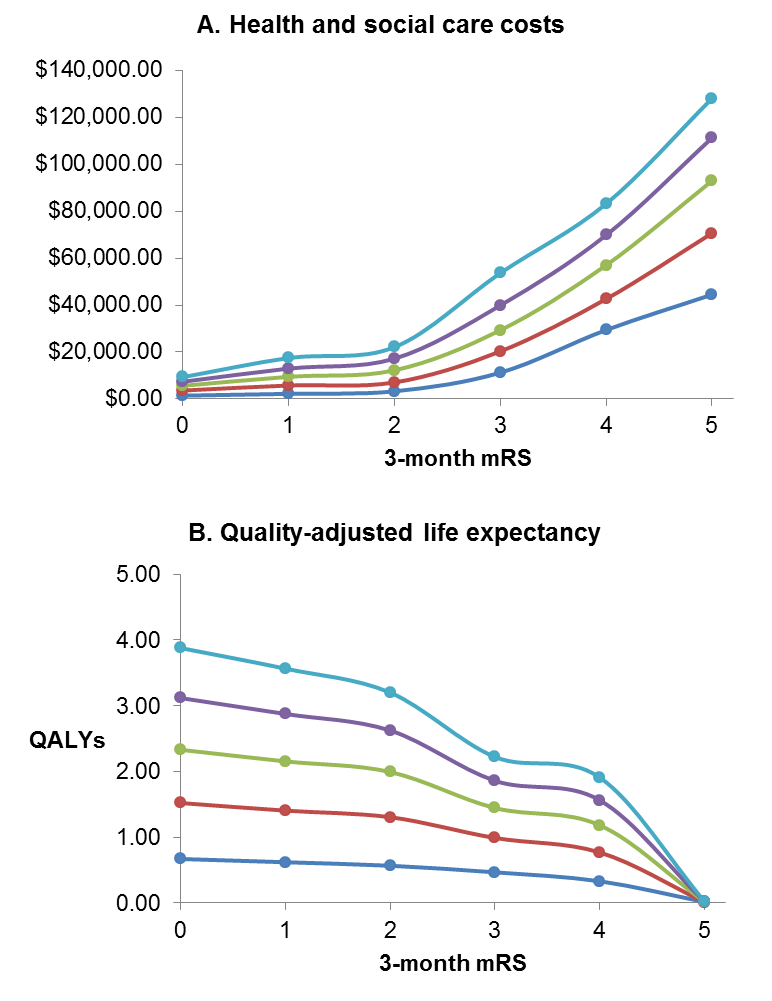
**

**Web Appendix 25.** Estimated (A) 5-year health and social care costs and (B) quality-adjusted life expectancy (in quality-adjusted life-years, QALYs) for 1-year (dark blue), 2-years (red), 3-years (green), 4-years (purple), and 5-years (light blue) post-stroke for 3-month survivors of ischaemic stroke, stratified by 3-month mRS, restricted to those aged <75 years (n=669).

**
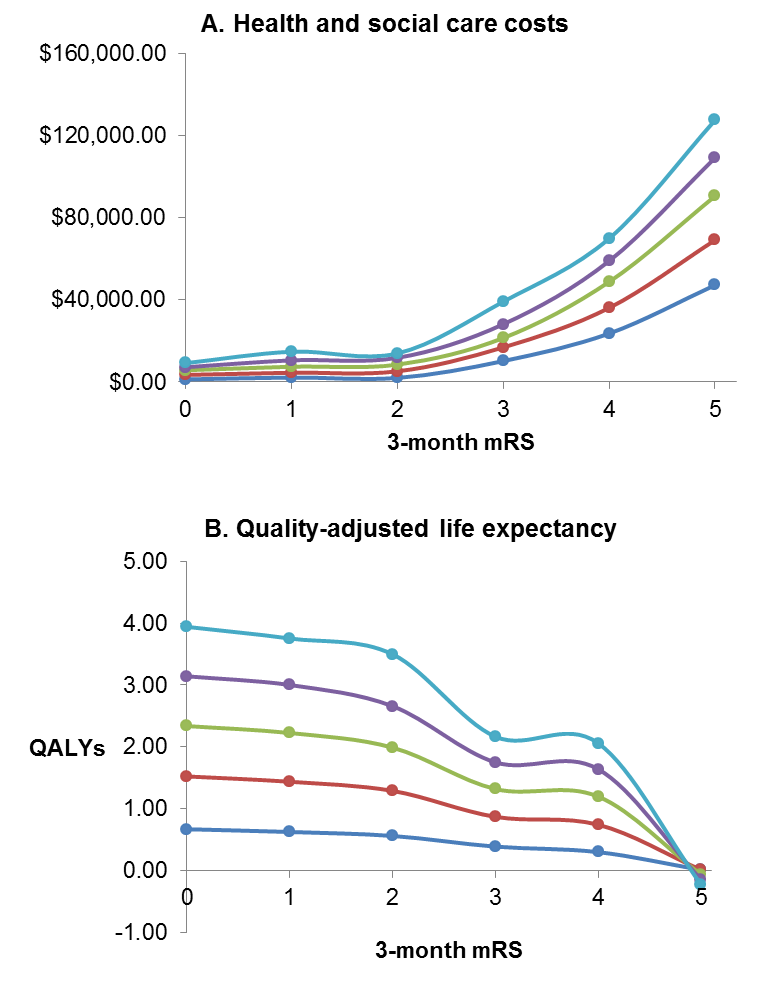
**

**Web Appendix 26.** Estimated (A) 5-year health and social care costs and (B) quality-adjusted life expectancy (in quality-adjusted life-years, QALYs) for 1-year (dark blue), 2-years (red), 3-years (green), 4-years (purple), and 5-years (light blue) post-stroke for 3-month survivors of ischaemic stroke, stratified by 3-month mRS, restricted to those aged >75 years (n=733).

**
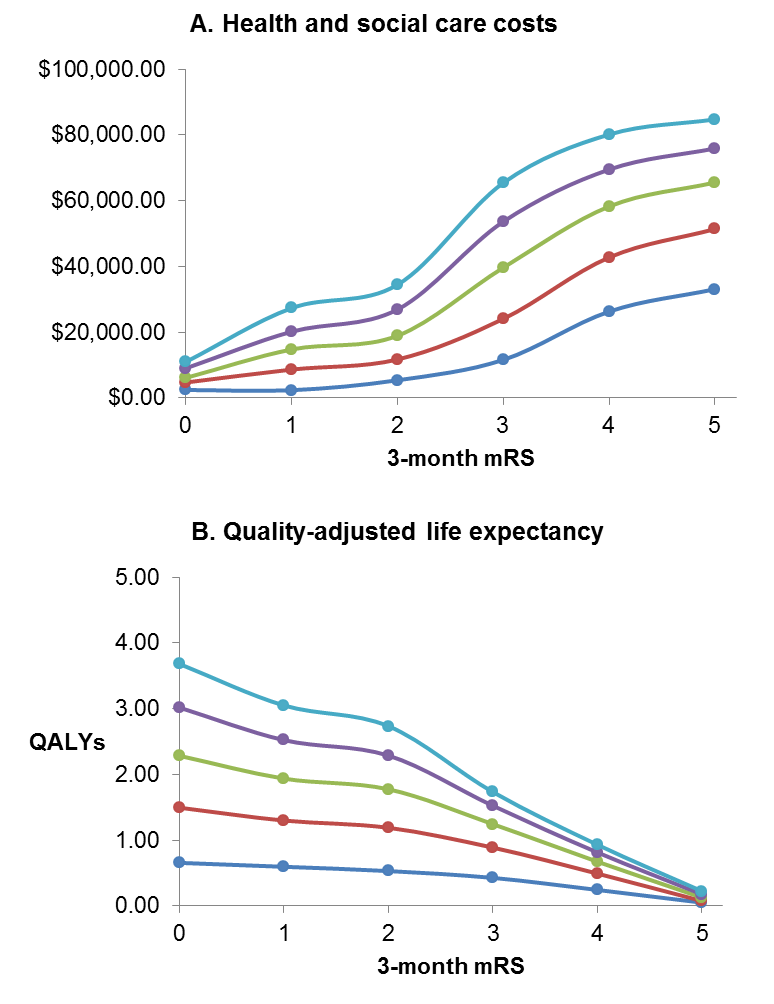
**

**Web Appendix 27.** Estimated (A) 5-year health and social care costs and (B) quality-adjusted life expectancy (in quality-adjusted life-years, QALYs) for 1-year (dark blue), 2-years (red), 3-years (green), 4-years (purple), and 5-years (light blue) post-stroke for 3-month survivors of ischaemic stroke, stratified by 3-month mRS, restricted to male sex (n=748).

**
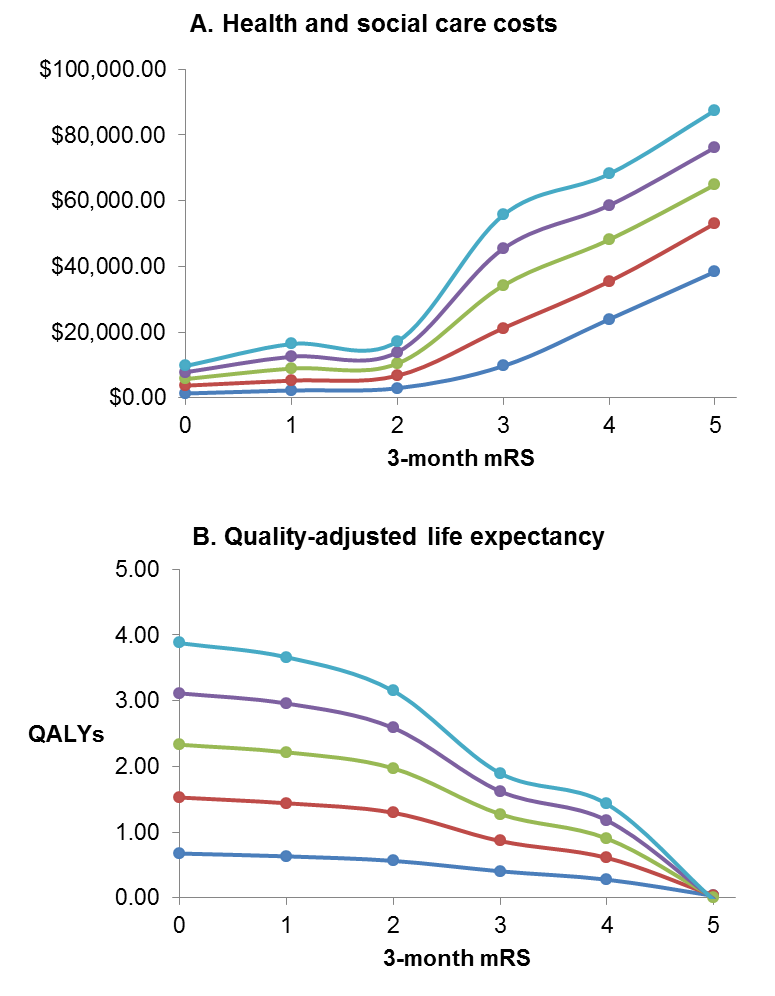
**

**Web Appendix 28.** Estimated (A) 5-year health and social care costs and (B) quality-adjusted life expectancy (in quality-adjusted life-years, QALYs) for 1-year (dark blue), 2-years (red), 3-years (green), 4-years (purple), and 5-years (light blue) post-stroke for 3-month survivors of ischaemic stroke, stratified by 3-month mRS, restricted to female sex (n=655).

**
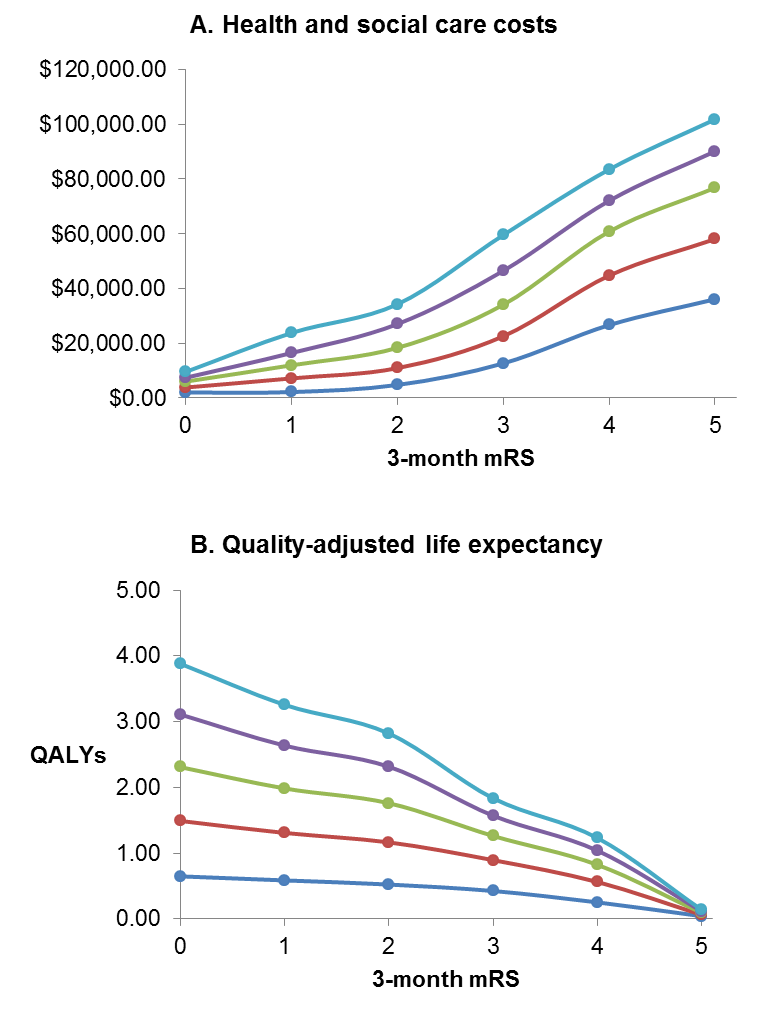
**

**Web Appendix 29. Comparison of 5-year quality-adjusted life expectancies (QALE weights) for 3-month survivors of ischaemic stroke, grouped by 3-month mRS, in: all patients, excluding premorbid mRS>2, excluding premorbid mRS>1, age<75, age>75, men, and women.** All QALEs are presented with two decimal places and alongside the number of individuals contributing to that estimate. Below the QALEs for the 5 subgroups, the difference compared to the estimated QALE for the overall cohort is presented (∆QALE = QALE for group – QALE for all patients). ∆QALEs >0.50 or <-0.50, representing 10% of the maximal 5-year QALE of 5.00, are flagged with *.

|  | **All Patients** | | **Excluding Pre-morbid mRS>2** | | **Excluding Pre-morbid mRS>1** | | **Age <75 years** | | **Age >75 years** | | **Men** | | **Women** | |
| --- | --- | --- | --- | --- | --- | --- | --- | --- | --- | --- | --- | --- | --- | --- |
| **3-month mRS** | **QALE** | **N** | **QALE**  **(∆QALE)** | **N** | **QALE**  **(∆QALE)** | **N** | **QALE**  **(∆QALE)** | **N** | **QALE**  **(∆QALE)** | **N** | **QALE**  **(∆QALE)** | **N** | **QALE**  **(∆QALE)** | **N** |
|  |  |  |  |  |  |  |  |  |  |  |  |  |  |  |
| **0** | 3.88 | 132 | 3.88  (0.00) | 132 | 3.88  (0.00) | 128 | 3.94  (0.06) | 96 | 3.68  (-0.20) | 36 | 3.88  (0.00) | 87 | 3.88  (0.00) | 45 |
| **1** | 3.49 | 379 | 3.51  (0.02) | 378 | 3.57  (0.08) | 349 | 3.75  (0.26) | 239 | 3.05  (-0.44) | 140 | 3.66  (0.17) | 217 | 3.26  (-0.23) | 162 |
| **2** | 3.01 | 276 | 3.03  (0.02) | 265 | 3.20  (0.19) | 217 | 3.50  (0.49) | 137 | 2.74  (-0.27) | 139 | 3.15  (0.14) | 158 | 2.82  (-0.19) | 118 |
| **3** | 1.87 | 219 | 2.05  (0.18) | 139 | 2.22  (0.35) | 94 | 2.17  (0.30) | 69 | 1.73  (-0.14) | 150 | 1.90  (0.03) | 111 | 1.84  (-0.03) | 108 |
| **4** | 1.30 | 112 | 1.71  (0.41) | 73 | 1.91  (0.61*) | 56 | 2.06  (0.76*) | 39 | 0.93  (-0.37) | 73 | 1.43  (0.13) | 43 | 1.23  (-0.07) | 69 |
| **5** | 0.06 | 49 | 0.02  (-0.04) | 34 | 0.02  (-0.04) | 27 | -0.22  (-0.28) | 14 | 0.23  (0.17) | 35 | -0.04  (-0.10) | 16 | 0.14  (0.08) | 33 |
| **Total N** |  | 1167 |  | 1021 |  | 871 |  | 594 |  | 573 |  | 632 |  | 535 |

**Web Appendix 30. Effect sizes estimated using 5-year probability-, cost-, and QALE-weights for dichotomous analysis of the mRS in recent thrombectomy trials, with a 0-1/2-5 dichotomy, compared to the odds ratio (OR) from binary logistic regression.** Values are presented as means with 95% confidence intervals. All P-values are <0.0001 unless otherwise indicated. Non-significant P-values>0.05 are indicated with an asterisk (*). PrDDI = probability of death/dementia/institutionalization, Delta = difference between treatment and control groups (all positive values favourable), Rx = treatment group, Ctrl = control group.

| **Trials** | **OR**  **(mRS 0-1)** | **Mean 5-year PrDDI (Rx)** | **Mean 5-year PrDDI (Ctrl)** | **Delta 5-year PrDDI** | **Mean 5-year Costs (Rx)** | **Mean 5-year Costs (Ctrl)** | **Delta 5-year Costs** | **Mean 5-year QALE (Rx)** | **Mean 5-year QALE (Ctrl)** | **Delta 5-year QALE** |
| --- | --- | --- | --- | --- | --- | --- | --- | --- | --- | --- |
| All | **2.22**  (1.79-2.76) | 0.59  (0.57-0.60) | 0.65  (0.64-0.67) | **0.07**  (0.05-0.09) | $35,909  (34,545-37,273) | $39,100  (37,707-40,494) | **$3,192**  (1,243-5,141)  P=0.0013 | 2.22  (2.15-2.29) | 1.95  (1.89-2.02) | **0.27**  (0.18-0.37) |
| DEFUSE-3 | **2.53**  (1.16-5.55)  P=0.02 | 0.60  (0.55-0.64) | 0.69  (0.64-0.74) | **0.10**  (0.03-0.16)  P=0.0058 | $37,398  (32,793-42,003) | $36,347  (31,192-41,503) | **-$1,051**  (-7,909-5,807)  P=0.76* | 2.20  (1.97-2.43) | 1.75  (1.51-1.99) | **0.45**  (0.12-0.77)  P=0.0077 |
| DAWN | **4.66**  (2.10-10.3) | 0.59  (0.54-0.64) | 0.68  (0.64-0.72) | **0.09**  (0.02-0.15)  P=0.0067 | $32,740  (28,339-37,142) | $41,587  (37,106-46,067) | **$8,847**  (2,598-15,095)  P=0.0057 | 2.19  (1.95-2.42) | 1.86  (1.67-2.06) | **0.32**  (0.02-0.63) |
| THRACE | **1.37**  (0.90-2.09)  P=0.14* | 0.55  (0.52-0.59) | 0.58  (0.55-0.62) | **0.03**  (-0.02-0.08)  P=0.19* | $35,205  (32,176-38,234) | $37,014  (33,958-40,070) | **$1,809**  (-2,481-6,099)  P=0.41* | 2.37  (2.22-2.53) | 2.25  (2.09-2.40) | **0.13**  (-0.09-0.35)  P=0.24* |
| MR CLEAN | **2.01**  (1.07-3.77)  P=0.03 | 0.68  (0.65-0.70) | 0.70  (0.68-0.72) | **0.03**  (-0.01-0.06)  P=0.14* | $38,915  (35,885-41,944) | $40,461  (37,636-43,286) | **$1,546**  (-2,586-5,678)  P=0.46* | 1.84  (1.71-1.98) | 1.74  (1.62-1.86) | **0.11**  (-0.07-0.29)  P=0.25* |
| ESCAPE | **2.62**  (1.54-4.44) | 0.54  (0.50-0.58) | 0.65  (0.61-0.68) | **0.11**  (0.05-0.16)  P=0.0001 | $36,071  (32,793-39,349) | $37,864  (34,083-41,646) | **$1,793**  (-3,167-6,754)  P=0.48* | 2.44  (2.27-2.60) | 1.97  (1.79-2.15) | **0.47**  (0.22-0.71)  P=0.0002 |
| REVASCAT | 2.22  (1.06-4.63)  P=0.034 | 0.62  (0.57-0.67) | 0.65  (0.61-0.69) | **0.04**  (-0.03-0.10)  P=0.25* | $35,708  (31,181-40,236) | $41,711  (37,439-45,982) | **$6,002**  (-186-12,189)  P=0.057* | 2.08  (1.85-2.31) | 1.97  (1.78-2.16) | **0.11**  (-0.19-0.41)  P=0.46* |
| SWIFT  PRIME | **3.13**  (1.63-6.00)  P=0.001 | 0.51  (0.46-0.56) | 0.62  (0.57-0.66) | **0.11**  (0.04-0.17)  P=0.002 | $33,787  (29,569-38,006) | $40,616  (36,179-45,052) | **$6,828**  (750-12,906)  P=0.028 | 2.55  (2.33-2.77) | 2.12  (1.92-2.33) | **0.43**  (0.13-0.72)  P=0.005 |
| EXTEND  IA | **2.65**  (0.99-7.11)  P=0.054* | 0.47  (0.39-0.56) | 0.61  (0.51-0.70) | **0.13**  (0.01-0.25)  P=0.037 | $30,888  (23,828-37,947) | $33,230 (25,147-41,314) | **$2,343**  (-8,195-12,881)  P=0.66* | 2.69  (2.31-3.07) | 2.11  (1.68-2.54) | **0.58**  (0.02-1.14)  P=0.043 |

**Web Appendix 31. Effect sizes estimated using 5-year probability-, cost-, and QALE-weights for dichotomous analysis of the mRS in recent thrombectomy trials, with a 0-2/3-5 dichotomy, compared to the odds ratio (OR) from binary logistic regression.** Values are presented as means with 95% confidence intervals. All P-values are <0.0001 unless otherwise indicated. Non-significant P-values>0.05 are indicated with an asterisk (*). PrDDI = probability of death/dementia/institutionalization, Delta = difference between treatment and control groups (all positive values favourable), Rx = treatment group, Ctrl = control group.

| **Trials** | **OR**  **(mRS 0-2)** | **Mean 5-year PrDDI (Rx)** | **Mean 5-year PrDDI (Ctrl)** | **Delta 5-year PrDDI** | **Mean 5-year Costs (Rx)** | **Mean 5-year Costs (Ctrl)** | **Delta 5-year Costs** | **Mean 5-year QALE (Rx)** | **Mean 5-year QALE (Ctrl)** | **Delta 5-year QALE** |
| --- | --- | --- | --- | --- | --- | --- | --- | --- | --- | --- |
| All | **2.15**  (1.67-2.76) | 0.59  (0.57-0.61) | 0.69  (0.68-0.71) | **0.10**  (0.08-0.12) | $36,433  (34,713-38,154) | $44,349  (42,491-46,206) | **$7,916**  (5,385-10,446) | 2.16  (2.08-2.23) | 1.73  (1.66-1.80) | **0.43**  (0.33-0.53) |
| DEFUSE-3 | **6.25**  (1.99-19.7)  P=0.002 | 0.60 (0.55-0.66) | 0.76  (0.71-0.80) | **0.04**  (0.08-0.23) | $38,506  (32,559-44,454) | $44,729  (37,963-51,494) | **$6,223**  (-2,713-15,158)  P=0.17 | 2.11  (1.86-2.37) | 1.42  (1.19-1.64) | **0.69**  (0.36-1.04)  P=0.0001 |
| DAWN | **7.04**  (2.26-21.9)  P=0.0001 | 0.59  (0.54-0.65) | 0.76  (0.72-0.80) | **0.17**  (0.10-0.23) | $33,156  (27,798-38,514) | $51,838  (45,854-57,822) | **$18,682**  (10,720-26,644) | 2.12  (1.87-2.38) | 1.46  (1.27-1.65) | **0.67**  (0.35-0.98)  P=0.0001 |
| THRACE | **1.55**  (1.05-2.30)  P=0.029 | 0.56  (0.52-0.60) | 0.61  (0.57-0.65) | **0.05**  (0.002-0.11)  P=0.044 | $35,677  (31,885-39,468) | $40,338  (36,335-44,340) | **$4,661**  (-837-10,160)  P=0.096* | 2.30  (2.13-2.47) | 2.08  (1.91-2.24) | **0.23**  (-0.01-0.46) |
| MR CLEAN | **2.04**  (1.36-3.07)  P=0.001 | 0.67  (0.64-0.71) | 0.74  (0.71-0.76) | **0.07**  (0.02-0.11)  P=0.003 | $39,512  (35,570-43,453) | $45,756  (41,970-49,542) | **$6,244**  (772-11,706)  P=0.025 | 1.79  (1.63-1.95) | 1.52  (1.39-1.65) | **0.27**  (0.07-0.48)  P=0.009 |
| ESCAPE | **2.78**  (1.74-4.44) | 0.55  (0.51-0.59) | 0.68 (0.64-0.72) | **0.13**  (0.08-0.19) | $36,629  (32,473-40,785) | $42,541  (37,556-47,526) | **$5,911**  (-508-12,331)  P=0.07* | 2.36  (2.18-2.54) | 1.76  (1.57-1.96) | **0.60**  (0.33-0.86) |
| REVASCAT | 1.98  (1.11-3.53)  P=0.021 | 0.62  (0.56-0.67) | 0.68  (0.63-0.73) | **0.07**  (-0.006-0.14)  P=0.072* | $35,866  (30,202-41,531) | $45,976  (40,119-51,833) | **$10,109**  (2,010-18,209)  P=0.015 | 2.03  (1.78-2.29) | 1.78  (1.56-2.00) | **0.25**  (-0.08-0.58)  P=0.14* |
| SWIFT  PRIME | **2.75**  (1.53-4.94)  P=0.001 | 0.52  (0.46-0.60) | 0.64  (0.59-0.69) | **0.12**  (0.05-0.20)  P=0.001 | $33,974  (28,804-39,143) | $44,076  (38,044-50,108) | **$10,102**  (2,236-17,968)  P=0.012 | 2.48  (2.25-2.72) | 1.96  (1.72-2.20) | **0.52**  (0.19-0.85)  P=0.002 |
| EXTEND  IA | **3.75**  (1.38-10.2)  P=0.009 | 0.46  (0.38-0.55) | 0.64  (0.54-0.73) | **0.17**  (0.05-0.30)  P=0.009 | $28,616  (20,873-36,358) | $36,659  (26,372-46,935) | **$8,043**  (-4,591-20,677)  P=0.21* | 2.70  (2.31-3.09) | 1.94  (1.49-2.39) | **0.76**  (0.18-1.35)  P=0.011 |

**Web Appendix 32. Effect sizes for 5-year probability of death/dementia/institutionalization, costs, and QALE, estimated using a “linear” ordinal analysis (assuming equal differences for each mRS increment), of the mRS in recent thrombectomy trials, compared to the common odds ratio (cOR) from ordinal logistic regression.** Values are presented as means with 95% confidence intervals. All P-values are <0.0001 unless otherwise indicated. Non-significant P-values>0.05 are indicated with an asterisk (*). PrDDI = probability of death/dementia/institutionalization, Delta = difference between treatment and control groups (all positive values favourable), Rx = treatment, Ctrl = control group.

| **Trials** | **cOR**  **(Ordinal)** | **Mean 5-year PrDDI (Rx)** | **Mean 5-year PrDDI (Ctrl)** | **Delta 5-year PrDDI** | **Mean 5-year Costs (Rx)** | **Mean 5-year Costs (Ctrl)** | **Delta 5-year Costs** | **Mean 5-year QALE (Rx)** | **Mean 5-year QALE (Ctrl)** | **Delta 5-year QALE** |
| --- | --- | --- | --- | --- | --- | --- | --- | --- | --- | --- |
| All | **2.01**  (1.72-2.34) | 0.58  (0.56-0.59) | 0.67  (0.66-0.69) | **0.09**  (0.07-0.12) | $42,251  (40,439-44,064) | $50,671  (48,628-52,715) | **$8,420**  (5,689-11,151) | 2.03  (1.95-2.11) | 1.57  (1.50-1.64) | **0.46**  (0.36-0.56) |
| DEFUSE-3 | **2.77**  (1.63-4.71) | 0.59  (0.54-0.64) | 0.73  (0.68-0.78) | **0.14**  (0.07-0.21)  P=0.0002 | $44,867  (38,559-51,174) | $49,578  (41,783-57,373) | **$4,711**  (-5,226-14,649)  P=0.35* | 1.97  (1.72-2.22) | 1.31  (1.07-1.55) | **0.66**  (0.31-1.01)  P=0.0002 |
| DAWN | **2.83**  (1.71-4.68) | 0.59  (0.54-0.64) | 0.73  (0.69-0.77) | **0.14**  (0.08-0.21) | $39,140  (33,384-44,896) | $58,170  (51,275-65,065) | **$19,030**  (10,152-27,909) | 1.99  (1.74-2.23) | 1.30  (1.10-1.50) | **0.69**  (0.37-1.01) |
| THRACE | **1.39**  (0.99-1.97)  P=0.059* | 0.54  (0.51-0.58) | 0.59  (0.55-0.62) | **0.05**  (-0.004-0.09)  P=0.074* | $41,196  (37,094-45,299) | $45,545  (41,272-49,818) | **$4,349**  (-1,558-10,256)  P=0.15* | 2.19  (2.02-2.37) | 1.97  (1.80-2.14) | **0.22**  (-0.02-0.46)  P=0.073* |
| MR CLEAN | **1.65**  (1.20-2.26)  P=0.002 | 0.66  (0.63-0.69) | 0.72  (0.70-0.75) | **0.06**  (0.02-0.10)  P=0.002 | $46,256  (42,264-50,249) | $52,921  (48,880-56,962) | **$6,664**  (966-12,362)  P=0.022 | 1.62  (1.48-1.76) | 1.33  (1.21-1.45) | **0.29**  (0.11-0.48)  P=0.002 |
| ESCAPE | **2.58**  (1.73-3.86) | 0.53  (0.49-0.57) | 0.66  (0.62-0.71) | **0.13**  (0.08-0.19) | $42,139  (37,678-46,599) | $48,755  (43,238-54,272) | **$6,616**  (-390-13,622)  P=0.064* | 2.25  (2.07-2.44) | 1.61  (1.42-1.81) | **0.64**  (0.37-0.91) |
| REVASCAT | **1.57**  (0.97-2.55)  P=0.067* | 0.61  (0.56-0.66) | 0.57  (0.63-0.72) | **0.06**  (0.007-0.13)  P=0.076* | $42,732  (36,654-48,812) | $53,952  (47,404-60,500) | **$11,219**  (2,338-20,100)  P=0.014 | 1.86  (1.62-2.11) | 1.57  (1.35-1.79) | **0.29**  (-0.03-0.62)  P=0.077* |
| SWIFT  PRIME | **2.55**  (1.53-4.27) | 0.50  (0.45-0.55) | 0.63  (0.58-0.68) | **0.13**  (0.06-0.20)  P=0.0005 | $38,865  (33,387-44,343) | $50,929  (44,364-57,493) | **$12,064**  (3,604-20,524)  P=0.0054 | 2.40  (2.16-2.64) | 1.79  (1.55-2.03) | **0.61**  (0.27-0.94)  P=0.0005 |
| EXTEND  IA | **3.22**  (1.35-7.68)  P=0.008 | 0.44  (0.35-0.51) | 0.62  (0.52-0.72) | **0.18**  (0.05-0.31)  P=0.0055 | $31,143  (23,619-38,667) | $41,424  (29,481-53,367) | **$10,281**  (-3,579-24,141)  P=0.14* | 2.71  (2.33-3.10) | 1.85  (1.37-2.33) | **0.87**  (0.26-1.47)  P=0.0054 |

**Web Appendix 33. Effect sizes estimated using a weighted ordinal analysis with 5-year probability-, cost-, and QALE-weights for the mRS in recent thrombectomy trials, compared to the common odds ratio (cOR) from ordinal logistic regression.** Values are presented as means with 95% confidence intervals. All P-values are <0.0001 unless otherwise indicated. Non-significant P-values>0.05 are indicated with an asterisk (*). PrDDI = probability of death/dementia/institutionalization, Delta = difference between treatment and control groups (all positive values favourable), Rx = treatment group, Ctrl = control group.

| **Trials** | **cOR**  **(Ordinal)** | **Mean 5-year PrDDI (Rx)** | **Mean 5-year PrDDI (Ctrl)** | **Delta 5-year PrDDI** | **Mean 5-year Costs (Rx)** | **Mean 5-year Costs (Ctrl)** | **Delta 5-year Costs** | **Mean 5-year QALE (Rx)** | **Mean 5-year QALE (Ctrl)** | **Delta 5-year QALE** |
| --- | --- | --- | --- | --- | --- | --- | --- | --- | --- | --- |
| All | **2.01**  (1.72-2.34) | 0.59  (0.57-0.61) | 0.70  (0.69-0.72) | **0.11**  (0.09-0.14) | $36,301  (34,459-38,142) | $46,494  (44,399-48,588) | **$10,193**  (7,405-12,981) | 2.11  (2.03-2.19) | 1.56  (1.48-1.64) | **0.55**  (0.43-0.66) |
| DEFUSE-3 | **2.77**  (1.63-4.71) | 0.60  (0.55-0.66) | 0.77  (0.71-0.82) | **0.16**  (0.08-0.24)  P=0.0001 | $39,024  (32,540-45,507) | $47,216  (39,432-54,999) | **$8,192**  (-1,851-18,234)  P=0.11* | 2.03  (1.75-2.32) | 1.24  (0.97-1.50) | **0.80**  (0.41-1.18)  P=0.0001 |
| DAWN | **2.83**  (1.71-4.68) | 0.59  (0.53-0.65) | 0.77  (0.73-0.82) | **0.18**  (0.11-0.25) | $33,357  (27,572-39,141) | $55,668  (48,688-62,648) | **$22,312**  (13,353-31,270) | 2.07  (1.79-2.35) | 1.21  (0.99-1.44) | **0.86**  (0.50-1.22) |
| THRACE | **1.39**  (0.99-1.97)  P=0.059* | 0.55  (0.51-0.59) | 0.60  (0.56-0.64) | **0.05**  (-0.003-0.11)  P=0.064* | $35,348  (31,193-39,504) | $40,546  (36,182-44,910) | **$5,198**  (-812-11,207)  P=0.090* | 2.28  (2.09-2.47) | 2.04  (1.86-2.23) | **0.24**  (-0.03-0.50)  P=0.076* |
| MR CLEAN | **1.65**  (1.20-2.26)  P=0.002 | 0.68  (0.70-0.74) | 0.76  (0.73-0.78) | **0.07**  (0.03-0.11)  P=0.001 | $40,275  (36,187-44,363) | $48,641  (44,484-52,798) | **$8,366**  (2,516-14,215)  P=0.005 | 1.69  (1.53-1.85) | 1.32  (1.18-1.46) | **0.37**  (0.16-0.58)  P=0.001 |
| ESCAPE | **2.58**  (1.73-3.86) | 0.54  (0.50-0.58) | 0.69  (0.65-0.73) | **0.15**  (0.09-0.21) | $36,085  (31,528-40,642) | $44,505  (38,868-50,139) | **$8,418**  (1,262-15,575)  P=0.02 | 2.33  (2.12-2.54) | 1.61  (1.39-1.83) | **0.72**  (0.42-1.02) |
| REVASCAT | **1.57**  (0.97-2.55)  P=0.067* | 0.63  (0.57-0.68) | 0.71  (0.66-0.76) | **0.08**  (0.005-0.16)  P=0.038 | $36,693  (30,485-42,902) | $42,267  (42,420-56,114) | **$12,574**  (3,387-21,761)  P=0.0075 | 1.91  (1.63-2.19) | 1.52  (1.26-1.78) | **0.39**  (0.01-0.77)  P=0.044 |
| SWIFT  PRIME | **2.55**  (1.53-4.27) | 0.50  (0.44-0.56) | 0.65  (0.60-0.71) | **0.15**  (0.07-0.23)  P=0.0002 | $32,739  (27,232-38,245) | $45,824  (38,978-52,670) | **$13,085**  (4,400-21,770)  P=0.0033 | 2.52  (2.26-2.77) | 1.80  (1.53-2.08) | **0.71**  (0.34-1.09)  P=0.0002 |
| EXTEND  IA | **3.22**  (1.35-7.68)  P=0.008 | 0.43  (0.34-0.52) | 0.63  (0.52-0.74) | **0.20**  (0.06-0.34)  P=0.0054 | $24,521  (17,719-31,323) | $37,515  (25,544-49,488) | **$12,995**  (-525-26,515)  P=0.059* | 2.85  (2.46-3.25) | 1.85  (1.33-2.38) | **1.00**  (0.36-1.65)  P=0.003 |
